# Prevalence and risk factors of perinatal depression among mothers and fathers in Pakistan: a systematic review and meta-analysis

**DOI:** 10.1080/21642850.2024.2383468

**Published:** 2024-08-09

**Authors:** Zahra Ali Padhani, Rehana A. Salam, Komal Abdul Rahim, Samra Naz, Asma Zulfiqar, Zahid Ali Memon, Salima Meherali, Maria Atif, Zohra S. Lassi

**Affiliations:** a School of Public Health, Faculty of Health and Medical Sciences, University of Adelaide, Adelaide, Australia; b Robinson Research Institute, Faculty of Health and Medical Sciences, University of Adelaide, Adelaide, Australia; c Centre of Research Excellence, Melanoma Institute Australia, University of Sydney, Sydney, Australia; d Internal Medicine, Aga Khan University, Karachi, Pakistan; e Dean’s Office, Medical College, Aga Khan University, Karachi, Pakistan; f Australian Institute for Machine Learning, University of Adelaide, Adelaide, Australia; g Institute for Social Science Research, University of Queensland, Queensland, Australia; h Institute for Global Health and Development, Aga Khan University, Karachi, Pakistan; i Department of Community Health Sciences, Aga Khan University, Karachi, Pakistan; j College of Health Sciences, Faculty of Nursing, University of Alberta, Edmonton Clinic Health Academy, Edmonton, Canada; k School of Public Health, Dow University of Health Sciences, Karachi, Pakistan

**Keywords:** Perinatal depression, antenatal depression, postnatal depression, maternal depression, paternal depression

## Abstract

**Background:**

Perinatal mental health issues affect approximately 10% of women in high-income countries and 30% in low- or middle-income countries. This review aims to determine the prevalence of perinatal depression among mothers and fathers in Pakistan and identify associated risk factors.

**Methods:**

We conducted a systematic review and meta-analysis following the Preferred Reporting Items for Systematic Reviews and Meta-Analysis guidelines. We included quantitative studies on the prevalence or incidence of maternal and paternal perinatal depression, including antenatal or postnatal depression in Pakistan, with or without associated risk factors. We performed an electronic search, dual-title/abstract and full-text screening, and data extraction. Analysis was conducted on Revman and JBI SUMARI software. The quality of the included studies was assessed with the NHLBI tool. This review updated a previously published review that included 43 studies, with the last search date of 31st May 2019, now extended to literature published up to June 30, 2023.

**Results:**

Consistent with the previous review, our analysis of 61 studies indicated a pooled prevalence of 37% (95% confidence interval (CI): 30.6–43.6) for maternal antenatal depression. Postnatal depression at different time points, revealed rates of 34.2% (95% CI: 22.7–46.7), 40.9% (95% CI: 0–97.4), and 43.1% (95% CI: 24.4–62.9) at 3, 6 and 12 months, respectively. Paternal postnatal depression was observed at 40.5% (95% CI: 14.9–69) based on two studies. Risk factors for maternal perinatal depression include multiparity, contraceptive failure, inadequate antenatal care, pregnancy-induced hypertension, previous psychiatric illness, passive smoking, drug abuse, low socio-economic status, marital problems, family hardships, recent bereavement, housing difficulties, food insecurity, husband's illiteracy, his unemployment, and being blamed for child disability.

**Conclusion:**

The findings reveal a high prevalence of perinatal depression among mothers with very limited evidence of fathers residing in Pakistan, emphasising the need for prospective studies addressing mental health challenges.

**Registration:**

This review is registered on PROSPERO (CRD42023442581).

## Background

Perinatal depression is considered as a mood disorder that occurs during pregnancy (i.e. antenatal or prenatal depression) and weeks after childbirth (i.e. postnatal or postpartum depression (PPD)) (National Institute of Mental Health, 2023). The condition affects 20–25% of women in low- and middle-income countries (LMICs), posing significant risks during pregnancy and the first postnatal year (Fisher et al., 2012; Gelaye et al., 2016; Woody et al., 2017). It can lead to intense sadness, anxiety, hindered bonding, breastfeeding difficulties for mothers, and, in severe cases, harm to self and the infant (Cook et al., 2018). The level of despair can, at times, be so profound that some describe life as an overwhelming struggle, referring to it as a ‘death swamp’ contrary to the usual perception of birth as the most joyful phase of a mother's life (Corwin et al., 2003). If left untreated, it can lead to adverse birth outcomes such as low birth weight as well as hinder infants’ social, cognitive, and emotional development (Cummings & Davies, 1994; Rogers et al., 2020). These risk factors may include early-life stressors, limited social support, intimate partner violence, unintended pregnancy, and somatic symptoms during pregnancy (Dagher et al., 2021). Additionally, lower levels of education, unemployment, marital distress, and a history of mood disorders elevate the risk of maternal and paternal perinatal mood disorders. Identifying individuals at risk through targeted screening programs and implementing prevention and management protocols grounded in evidence-based interventions is imperative.

The global prevalence of postnatal depression among women varies widely according to the geographic regions with significantly higher rates in LMICs (Wang et al., 2021). In Pakistan, the prevalence of postnatal depression is reported to be 37%, which is among the highest in Asian countries (Atif et al., 2021). Traditionally, the discussions around birth-related matters have focused predominantly on women, leading to extensive data availability on maternal postnatal depression. However, recently there has been a growing recognition of postnatal depression in fathers, as a notable public health concern (Atif et al., 2022; Garthus-Niegel et al., 2020; Philpott & Corcoran, 2018; Wainwright et al., 2023).

Research findings indicate that the prevalence of paternal depression may vary ranging from 9.76% during the antenatal period to about 8.75% within the first year following birth (Paulson & Bazemore, 2010). However, a recent survey conducted by Atif et al. has reported a significantly higher prevalence of postnatal depression (23.5%) among Pakistani men (Atif et al., 2022). The variations in estimates may be affected by various factors, including the stigma surrounding mental health issues, variations in reporting methods, differences in how cognitive health variables are perceived, the absence of standardised practices for collecting data on maternal mental health, and the lack of a reliable, consistent reporting tool (Atif et al., 2023 Halbreich & Karkun, 2006; Ramadas & Kumar, 2016;). The lack of uniformity of data collection tools and procedures has led to significant variability in the prevalence rates of perinatal depression in Pakistan, thus challenging gauging the true magnitude of this issue (Atif et al., 2021). Given the variability in the prevalence of postnatal depression and limited evidence on the factors associated with perinatal depression among mothers and fathers, we aim to determine the prevalence of perinatal depression among mothers and fathers residing in Pakistan and identify its associated risk factors. This review will serve to consolidate and critically evaluate existing research on maternal and paternal postnatal depression in Pakistan, ultimately informing targeted interventions and policies to address this pressing public health concern.

## Methods

We conducted a systematic review and meta-analysis following the Preferred Reporting Items for Systematic Reviews and Meta-Analysis (PRISMA) guidelines (See Annex 1) (Moher et al., 2009; Page et al., 2021). This review is registered on PROSPERO (CRD42023442581) and constitutes an update of a review previously published by Atif et al. (2021). The original review’s search was conducted until 31^st^ May 2019 and included 43 studies. However, only one study from the original review was excluded in this updated version, as it recruited all women with PPD. In the current review, we have updated our search to include literature published up to 30 June 2023, thus including recently published papers and reports on the current prevalence of depression among mothers and fathers. Additionally, this review estimates the prevalence of maternal perinatal depression by pooling data on maternal antenatal, perinatal, and postnatal depression. Furthermore, we conducted a meta-analysis of risk factors contributing to perinatal depression among mothers and fathers residing in Pakistan. This aspect of our review is novel, representing an advancement since the last review.

In this review, we included quantitative studies reporting on the prevalence or incidence of maternal and paternal perinatal depression, including antenatal or postnatal depression in Pakistan, with or without its associated risk factors. For the studies reporting on the associated risk factors of perinatal depression, we only meta-analysed studies that compared risk factors between depressed and non-depressed participants. We excluded qualitative studies, case studies, case reports, editorials, opinion pieces, and grey literature. We only included studies that were published in the English language. Refer to Table 1 for eligibility criteria.

**Table 1.** Eligibility criteria.

	Eligibility criteria
Population	Mothers and fathers during pregnancy or in their post-partum period
Exposure	Studies reporting on the prevalence or incidence of maternal and paternal perinatal depression, including antenatal or postnatal depression in Pakistan, with or without its associated risk factors
Comparison	For the studies reporting on the associated risk factors of perinatal depression, we only meta-analysed studies that compared risk factors between depressed and non-depressed participants
Outcome	Prevalence of antenatal, postnatal, and perinatal depression among mothers and fathers and its associated risk factors
Study design	We included quantitative studies including cohort and cross-sectional studies and excluded reviews, qualitative studies, case studies, case reports, editorials, opinion pieces, and grey literature
Setting	Studies conducted in Pakistan only

A search strategy was constructed using the population, exposure, comparison, and outcome (PECO) criteria, including keywords and MeSH terms (See Annex 2). An electronic database search was conducted on Medline, PsycINFO, CINAHL, and EMBASE. Field experts were contacted, and Google Scholar was searched to identify any additional relevant studies. All the studies identified through the database search were imported into EndNote and exported to Covidence software for de-duplication, title/abstract, and full-text screening (COVIDENCE, 2020). Two reviewers conducted the title and abstract screening, followed by full-text screening. Following the full-text screening, data was extracted from each included study onto a standardised data extraction form in a pretested Excel sheet. All the conflicts were resolved through discussion or by contacting the third reviewer. Study authors were contacted to obtain missing data from the included studies. Data was extracted on study characteristics, setting (country, rural/urban), participants, type of depression, the instrument to measure depression, and risk factors associated with perinatal depression.

To assess the quality of the included studies, we used the NHLBI quality assessment tool for cohort, case–control, and cross-sectional studies (NHLBI, 2019). The studies were judged to be of low, moderate, and high quality according to the 14 criteria based on the research question, study population, participation rate, sample selection and sample size justification, exposure and outcome measurement, blinding, attrition rate, and confounding. For the quality assessment, the study was ranked as high-quality if it did not have a ‘no’ in any of the components. If the study had ‘no’ in one or two components, the quality was marked as moderate, and if the study had ‘no’ in more than two components, the quality was kept as low. For criterion number 10, which states, ‘Was the exposure assessed more than once over time?’ If the study did not assess the exposure at multiple time points as in a cross-sectional study, it was not downgraded on its quality.

We used Joanna Briggs Institute (JBI) SUMARI software to assess the pooled prevalence of antenatal/postnatal/perinatal depression using the Freeman-Tukey transformation (JBI SUMARI, 2019). We also used Review Manager (RevMan) software version 5.4 to study the risk factors associated with perinatal depression, using Mantel-Haenszel methods to calculate the weights for continuous and categorical outcomes (Review Manager, 2020). For dichotomous outcomes, we used odds ratio (OR), while for the continuous outcomes, mean difference (MD) or standardised mean difference (SMD), along with a 95% confidence interval (CI), were used. Statistical heterogeneity was assessed using τ^2^, I^2^, and the significance of the χ^2^ test; we also evaluated heterogeneity by visually inspecting forest plots. The I^2^ in the forest plot demonstrated the variation across the studies due to heterogeneity. The I^2^ of ≤25, 50, and ≥75% demonstrated low, medium and high levels of heterogeneity. We performed a random-effects analysis for all comparisons as the data was expected to be heterogeneous. We also planned to create funnel plots to explore possible publication biases among outcomes, however, we could not explore publication bias since none of the outcomes (on risk factors) included more than 10 studies.

## Results

**Figure 1.**
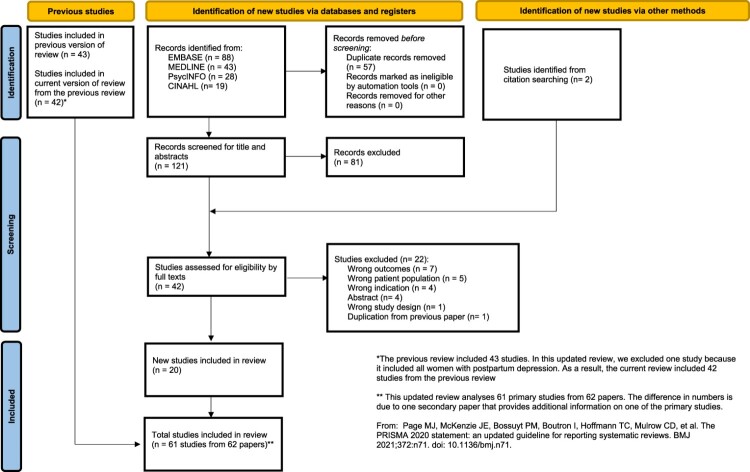
PRISMA Flow Diagram.

We ran an updated search and identified 121 studies to screen, of which 20 studies were found to be eligible for inclusion. Including the 42 studies from the previous review, this updated review reports findings from a total of 61 studies from 62 papers (See Annex 3) (Atif et al., 2022; Afridi et al., 2014; Ahmad & Khan, 2005; Ali et al., 2009; Ali et al., 2012; Asad et al., 2010; Ayyub et al., 2018; Brown et al., 2021; Chung et al., 2022; Ghaffar et al., 2017; Ghafoor et al., 2021; Gul et al., 2017; Gul et al., 2013; Habib & Ali, 2019; Habiba et al., 2020; Hamid et al., 2008; Hamirani et al., 2006; Humayun et al., 2013; Husain et al., 2006; Husain et al., 2011; Imran & Haider, 2010; Irum et al., 2022; Ishtiaque et al., 2020; Jabbar et al., 2022; Jamal et al., 2018; Kalar et al., 2012; Kalyani, 2001; Karmaliani et al., 2009; Kazi et al., 2006; Khalid, 1989; Khan et al., 2020; Khan et al., 2021; Khanam et al., 2011; Khanam et al., 2022; LeMasters et al., 2020; Maqbool et al., 2022; Maselko et al., 2018; Maselko et al., 2019; Masood et al., 2017; Mir et al., 2012; Muneer et al., 2009; Niaz et al., 2004; Noorullah et al., 2020; Premji et al., 2020; Rabia et al., 2017; Rahman & Creed, 2007; Rahman et al., 2003; Ramji et al., 2016; Riaz & Riaz, 2020; Sabir et al., 2019; Sadaf et al., 2011; Sadiq et al., 2016; Saeed et al., 2016; Shah & Lonergan, 2017; Shah et al., 2011; Shahid et al., 2022; Shaikh et al., 2011; Tariq et al., 2021; Zu et al., 2016; Waqas et al., 2015; Zahidie et al., 2011; Zareen et al., 2009) encompassing data from 23,838 women and 141 men (Figure 1). The majority of the studies were cross-sectional (*n* = 41), followed by cohort (*n* = 19) and case–control studies (*n* = 1). Studies were conducted between 2006 and 2023. Most of the studies were conducted in the metropolitan cities of Karachi (*n* = 16) and Lahore (*n* = 16). Most of the studies were conducted in urban settings (*n* = 29) (Afridi et al., 2014; Ahmad & Khan, 2005;; Ali et al., 2012 Asad et al., 2010; Atif et al., 2022; Ayyub et al., 2018; Gul et al., 2017; Hamid et al., 2008; Hamirani et al., 2006; Humayun et al., 2013; Husain et al., 2011; Imran & Haider, 2010; Jamal et al., 2018; Kalar et al., 2012; Kalyani, 2001; Karmaliani et al., 2009; Kazi et al., 2006; Khalid, 1989; Masood et al., 2017; Muneer et al., 2009; Niaz et al., 2004; Noorullah et al., 2020; Rabia et al., 2017; Ramji et al., 2016; Sadaf et al., 2011; Sadiq et al., 2016; Saeed et al., 2016; Shaikh et al., 2011; Zareen et al., 2009), while 10 were conducted in rural settings (Chung et al., 2022; Husain et al., 2006; Khan et al., 2021; LeMasters et al., 2020; Maselko et al., 2019; Mir et al., 2012; Rahman et al., 2003 Rahman & Creed, 2007;; Shah et al., 2011; Zahidie et al., 2011), five in sub-urban/peri-urban/urban slums (Ali et al., 2009; Gul et al., 2013; Khanam et al., 2011; Khanam et al., 2022; Zu et al., 2016), and five were conducted in both urban and rural settings (Ghaffar et al., 2017; Khan et al., 2020; Maqbool et al., 2022; Shah & Lonergan, 2017; Waqas et al., 2015). Twelve studies did not report on study settings (Brown et al., 2021; Ghafoor et al., 2021; Habib & Ali, 2019; Habiba et al., 2020; Irum et al., 2022; Ishtiaque et al., 2020; Jabbar et al., 2022; Premji et al., 2020; Riaz & Riaz, 2020; Sabir et al., 2019; Shahid et al., 2022; Tariq et al., 2021). Most studies were conducted in healthcare facility settings (*n* = 44) (Afridi et al., 2014; Ahmad & Khan, 2005;; Ali et al., 2012 Atif et al., 2022; Brown et al., 2021; Ghaffar et al., 2017; Ghafoor et al., 2021; Gul et al., 2013; Gul et al., 2017; Habiba et al., 2020; Hamid et al., 2008; Hamirani et al., 2006; Humayun et al., 2013; Husain et al., 2011; Imran & Haider, 2010; Irum et al., 2022; Ishtiaque et al., 2020; Jabbar et al., 2022; Jamal et al., 2018; Kalyani, 2001; Kazi et al., 2006; Khalid, 1989; Khan et al., 2020; Khanam et al., 2011; Maqbool et al., 2022; Masood et al., 2017; Mir et al., 2012; Muneer et al., 2009; Niaz et al., 2004; Noorullah et al., 2020; Premji et al., 2020; Rabia et al., 2017; Riaz & Riaz, 2020; Sabir et al., 2019; Sadaf et al., 2011; Sadiq et al., 2016; Saeed et al., 2016; Shah & Lonergan, 2017; Shahid et al., 2022; Shaikh et al., 2011; Tariq et al., 2021; Waqas et al., 2015; Zareen et al., 2009; Zu et al., 2016), while 15 studies were community-based (Ali et al., 2009; Asad et al., 2010; Ayyub et al., 2018; Chung et al., 2022; Husain et al., 2006; Kalar et al., 2012; Karmaliani et al., 2009; Khan et al., 2021; Khanam et al., 2022; Maselko et al., 2019; Rahman et al., 2003 Rahman & Creed, 2007;; Ramji et al., 2016; Shah et al., 2011; Zahidie et al., 2011), with one study in a rehabilitation centre (Habib & Ali, 2019), and one study did not report the details on the study settings (LeMasters et al., 2020). The sample size of the included studies ranged between 73–1369.

Almost all the studies focused on maternal perinatal depression except two studies that reported on paternal postnatal depression (Atif et al., 2022; Noorullah et al., 2020). Various tools were used to measure depression in the included studies. The most commonly used tool was the Edinburgh Postnatal Depression Scale (EPDS) used in 27 studies. Other tools used in the included studies were the Patient Health Questionnaire (PHQ) (*n* = 9), Hospital Anxiety and Depression Scale (*n* = 6), Aga Khan University Anxiety and Depression Scale (AKUDAS) (*n* = 5), Centre for Epidemiological Studies Depression scale (CESD) (*n* = 3), Hamilton Rating Scale (*n* = 3), Beck’s Depression Inventory (BDI) (*n* = 2). Other screening instruments used to assess depression were Goldberg’s Depression Scale, Depression Anxiety and Stress Scale, Zung Self-Rating Depression Scale, Siddique Shah Depression Scale, Pitt's questionnaire for purpureal depression, and Structured Clinical Interview for DSM disorders.

**Figure 2.**
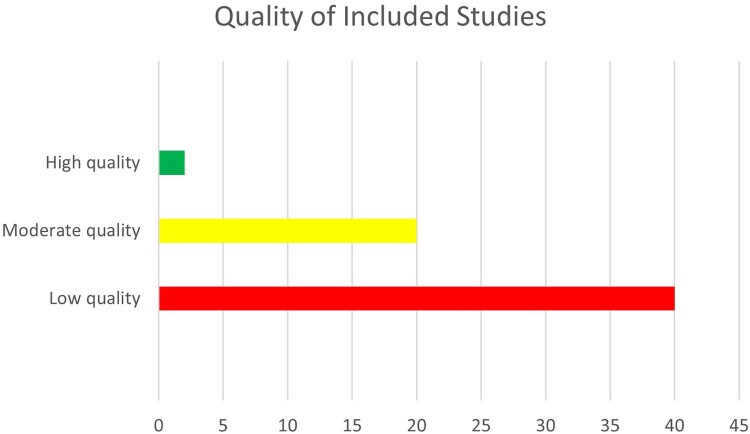
Quality of Included Studies.

Most of the included studies were judged to be of low quality. Cohort and cross-sectional studies were judged to be of low quality due to concerns with sample size and power estimations, small study duration to assess associations, lack of risk factor assessment over time, and insufficient measurement and adjustment of confounding variables (Figure 2). There was only one case–control study included in the review. The case–control study was judged to be of low quality because of a lack of information on sample size justification, blinding of assessors, and insufficient measurement and adjustment of confounding variables. The table in Annex 4 of the supplementary file details the risk of bias assessment. See Table 2 and Annex 5 and 6 for the risk factors associated with maternal and paternal perinatal depression.

**Table 2.** Risk factors associated with perinatal depression.

	Maternal			Paternal
Outcomes	Antenatal Depression	Postnatal Depression	Perinatal Depression (Combined)	Postnatal Depression
**Age**				
<30 years				**OR 11.25; 95% CI: 2.13, 59.38; *n* = 1**
> = 30 years				**OR 0.15; 95% CI: 0.03, 0.65; *n* = 1**
**Residence**				
Urban	OR 0.59; 95% CI: 0.24, 1.44; *n* = 1	**OR 0.48; 95% CI: 0.24, 0.97;***n* **= 1**	**OR 0.52; 95% CI: 0.30, 0.90;***n* **= 2; Heterogeneity: Chi² *P* = 0.73; I² = 0%**	
Rural	OR 1.69; 95% CI: 0.69, 4.11; *n* = 1	**OR 2.06; 95% CI: 1.03, 4.13;***n* **= 1**	**OR 1.91; 95% CI: 1.11, 3.30;***n* **= 2; Heterogeneity: Chi² *P* = 0.73; I² = 0%**	
**Education**				
Illiterate	**OR 2.01; 95% CI: 1.56, 2.58; *n* = 5; Heterogeneity: Chi² *P* = 0.76; I² = 0%**	OR 1.39; 95% CI: 0.73, 2.62; *n* = 6; Heterogeneity: Chi² *P* = 0.005; I² = 73%	**OR 1.56; 95% CI: 1.04, 2.34; *n* = 11; Heterogeneity: Chi² *P* < 0.00001; I² = 79%**	
Literate	**OR 0.48; 95% CI: 0.38, 0.62; *n* = 5; Heterogeneity: Chi² *P* = 0.92; I² = 0%**	OR 1.13; 95% CI: 0.61, 2.09; *n* = 5; Heterogeneity: Chi² *P* < 0.00001; I² = 85%	OR 0.72; 95% CI: 0.51, 1.03; *n* = 10; Heterogeneity: Chi² *P* = 0.003; I² = 63%	
**Education of family head/ husband**				
Illiterate	OR 0.97; 95% CI: 0.13, 6.98; *n* = 2; Heterogeneity: Chi² *P* = 0.15; I² = 52%	**OR 2.02; 95% CI: 1.24, 3.29;***n* **= 1**	**OR 1.83; 95% CI: 1.25, 2.67; *n* = 3; Heterogeneity: Chi² *P* = 0.31; I² = 15%**	
Literate	**OR 0.55; 95% CI: 0.36, 0.85; *n* = 2; Heterogeneity: Chi² *P* = 0.74; I² = 0%**			
**Husband’s Occupation**				
Unemployed		**OR 2.17; 95% CI: 1.19, 3.97;***n* **= 1**	**OR 2.34; 95% CI: 1.51, 3.63; *n* = 2; Heterogeneity: Chi² *P* = 0.72; I² = 0%**	**OR 12.67; 95% CI: 1.18, 136.44;***n* **= 1**
Employed			**OR 0.39; 95% CI: 0.21, 0.75;***n* **= 1**	**OR 0.08; 95% CI: 0.01, 0.85;***n* **= 1**
**Socioeconomic status**				
Upper	**OR 0.33; 95% CI: 0.18, 0.60; *n* = 1**		OR 0.60; 95% CI: 0.18, 1.98; *n *= 2; Heterogeneity: Chi² *P* = 0.01; I² = 85%	
Lower	OR 2.17; 95% CI: 0.66, 7.16; *n *= 1		**OR 2.67; 95% CI: 1.52, 4.67; *n *= 2; Heterogeneity: Chi² *P* = 0.70; I² = 0%**	
**Financially Independent**		**OR 0.45; 95% CI: 0.31, 0.67; *n *= 1**		
**Family debt/ hardships**				
Yes	**OR 2.15; 95% CI: 1.72, 2.70; *n *= 2; Heterogeneity: Chi² *P* = 0.59; I² = 0%**	OR 2.11; 95% CI: 0.84, 5.29; *n *= 2; Heterogeneity: Chi² *P* = 0.07; I² = 70%	**OR 2.20; 95% CI: 1.66, 2.90; *n *= 4; Heterogeneity: Chi² *P* = 0.30; I² = 18%**	**OR 8.70; 95% CI: 1.96, 38.65; *n *= 1**
No	**OR 0.51; 95% CI: 0.40, 0.64; *n *= 1**	**OR 0.30; 95% CI: 0.16, 0.58; *n *= 1**	**OR 0.43; 95% CI: 0.27, 0.68; *n *= 2; Heterogeneity: Chi² *P* = 0.14; I² = 53%**	**OR 0.11; 95% CI: 0.03, 0.51; *n *= 1**
**Food insecurity**	**OR 3.45; 95% CI: 2.59, 4.60; *n *= 2; Heterogeneity: Chi² *P* = 0.36; I² = 0%**			
**Pregnancy-related factors**				
**Planned Pregnancy**	**OR 0.35; 95% CI: 0.14, 0.85; *n *= 4; Heterogeneity: Chi² *P* < 0.00001; I² = 89%**		OR 0.57; 95% CI: 0.19, 1.70; *n *= 5; Heterogeneity: Chi² *P* < 0.00001; I² = 95%	
**History of previous C-sections**	**OR 0.50; 95% CI: 0.28, 0.91; *n *= 2; Heterogeneity: Chi² *P* = 0.59; I² = 0%**			
**Parity**				
Nulliparous	**OR 0.43; 95% CI: 0.26, 0.72; *n *= 4; Heterogeneity: Chi² *P* = 0.02; I² = 68%**	**OR 5.30; 95% CI: 1.31, 21.47; *n *= 1**	**OR 0.60; 95% CI: 0.38, 0.95; *n *= 6; Heterogeneity: Chi² *P* = 0.0001; I² = 80%**	
Multiparous	**OR 2.14; 95% CI: 1.14, 4.01; *n *= 5; Heterogeneity: Chi² *P* = 0.006; I² = 72%**	OR: 0.53; 95% CI: 0.11, 2.52; *n *= 4; Heterogeneity: Chi² *P* < 0.00001; I² = 97%	OR 1.16; 95% CI: 0.64, 2.09; *n *= 10; Heterogeneity: Chi² *P* < 0.00001; I² = 93%	
**Pregnancy duration**				
1st trimester	OR 0.36; 95% CI: 0.02, 5.27; *n *= 2; Heterogeneity: Chi² *P* < 0.00001; I² = 95%			
2nd trimester	**OR 0.52; 95% CI: 0.31, 0.86; *n *= 4; Heterogeneity: Chi² *P* = 0.06; I² = 59%**			
3rd trimester	OR 1.21; 95% CI: 0.60, 2.47; *n *= 4; Heterogeneity: Chi² *P* = 0.003; I² = 79%			
**Antenatal Care**				
Received		**OR 0.32; 95% CI: 0.11, 0.92; *n *= 1**		
Not Received		**OR 3.09; 95% CI: 1.09, 8.82; *n *= 1**		
**Contraception/ ever used contraception**	**OR 1.56; 95% CI: 1.07, 2.29; *n *= 2; Heterogeneity: Chi² *P* = 0.37; I² = 0%**		**OR 1.52; 95% CI: 1.08, 2.15; *n *= 3; Heterogeneity: Chi² *P* = 0.64; I² = 0%**	
**Separation from husband**				
Separated from husband	OR 7.53; 95% CI: 0.38, 147.73; *n *= 2; Heterogeneity: Chi² *P* = 0.04; I² = 76%			
Not Separated from husband	**OR 0.18; 95% CI: 0.04, 0.74; *n *= 2; Heterogeneity: Chi² *P* = 0.01; I² = 84%**			
**Marital problems**	**OR 7.57; 95% CI: 2.62, 21.85; *n *= 1**	OR 4.34; 95% CI: 0.34, 56.07; *n *= 2; Heterogeneity: Chi² *P* = 0.0006; I² = 91%	**OR 5.31; 95% CI: 1.21, 23.31; *n *= 3; Heterogeneity: Chi² *P* = 0.003; I² = 83%**	
**Quality of marital relationship**				
Fair	**OR 0.19; 95% CI: 0.08, 0.43; *n *= 1**			
Poor	**OR 5.40; 95% CI: 2.30, 12.65; *n *= 1**			
**Domestic Violence/IPV**				
Ye	OR 1.90; 95% CI: 0.56, 6.47; *n *= 5; Heterogeneity: Chi² *P* < 0.00001; I² = 91%	OR 13.38; 95% CI: 0.68, 264.05; *n *= 1	OR 2.26; 95% CI: 0.70, 7.30; *n *= 6; Heterogeneity: Chi² *P* < 0.00001; I² = 90%	
No	**OR 0.25; 95% CI: 0.07, 0.95; *n *= 5; Heterogeneity: Chi² *P* < 0.00001; I² = 92%**			
Unhappy/Problems with parents/in-laws	**OR 4.08; 95% CI: 2.36, 7.04; *n *= 2; Heterogeneity: Chi² *P* = 0.98; I² = 0%**	**OR 1.62; 95% CI: 1.01, 2.61; *n *= 2; Heterogeneity: Chi² *P* = 0.37; I² = 0%**	**OR 2.37; 95% CI: 1.32, 4.27; *n *= 4; Heterogeneity: Chi² *P* = 0.07; I² = 57%**	
Housing difficulties	**OR 2.81; 95% CI: 1.70, 4.63; *n *= 1**	OR 1.47; 95% CI: 0.51, 4.21; *n *= 1	**OR 2.41; 95% CI: 1.41, 4.13; *n *= 2; Heterogeneity: Chi² *P* = 0.28; I² = 16%**	
Feels Happy and Comfortable at Home		**OR 0.08; 95% CI: 0.02, 0.37; *n *= 1**		
Infant grandmother lives with family		**OR 0.49; 95% CI: 0.33, 0.74; *n *= 1**		
Daily physical help in childcare by at least 1 family member		**OR 0.38; 95% CI: 0.27, 0.54; *n *= 2; Heterogeneity: Chi² *P* = 0.47; I² = 0%**		
Prohibited to do household work during puerperium		**OR 0.32; 95% CI: 0.15, 0.68; n = 1**		
**Stressful life events**				
Death of some close relative (within 1 year)	**OR 1.97; 95% CI: 1.24, 3.14; *n *= 1**			
Loss of parents/mother	**OR 1.81; 95% CI: 1.11, 2.97; *n *= 1**	OR 2.26; 95% CI: 0.83, 6.15; *n *= 1	**OR 1.89; 95% CI: 1.22, 2.95; *n *= 2; Heterogeneity: Chi² *P* = 0.70; I² = 0%**	
**Sleep Disturbance**				
Yes				**OR 11.63; 95% CI: 2.54, 53.17; *n *= 1**
No				**OR 0.09; 95% CI: 0.02, 0.39; *n *= 1**
**Spouse Sleep Disturbance**				
Yes				**OR 5.36; 95% CI: 1.24, 23.10; *n *= 1**
No				**OR 0.19; 95% CI: 0.04, 0.80; *n *= 1**
**Spouse Depression status**				
Yes				**OR 11.25; 95% CI: 2.13, 59.38; *n *= 1**
No				**OR 0.09; 95% CI: 0.02, 0.47; *n *= 1**
**Other stressful life events**				
Yes	OR 2.05; 95% CI: 0.93, 4.51; n = 1			
No	**OR 0.05; 95% CI: 0.03, 0.11; *n *= 1**			
Satisfied with life	**OR 0.22; 95% CI: 0.06, 0.81; *n *= 1**			
Passive smoker	**OR 2.68; 95% CI: 1.73, 4.17; *n *= 1**			
**Coping mechanism**				
Drug abuse	**OR 2.48; 95% CI: 1.09, 5.62; *n *= 2; Heterogeneity: Chi² *P* = 0.44; I² = 0%**			
Regular physical activity	**OR 0.43; 95% CI: 0.20, 0.89; *n *= 1**			
Bereavement/Illness	OR 1.34; 95% CI: 0.88, 2.03; n = 1	OR 2.04; 95% CI: 0.98, 4.24; *n *= 1	**OR 1.49; 95% CI: 1.03, 2.13; *n *= 2; Heterogeneity: Chi² *P* = 0.33; I² = 0%**	
**Medical history**				
Depression/ psychiatric illness	OR 1.35; 95% CI: 0.43, 4.31; *n *= 3; Heterogeneity: Chi² *P* < 0.0001; I² = 90%			
Previous psychiatric illness/psychiatric drug use	**OR 2.57; 95% CI: 1.04, 6.35; *n *= 2; Heterogeneity: Chi² *P* = 0.14; I² = 54%**	**OR 7.14; 95% CI: 3.54, 14.37; *n *= 1**	**OR 3.42; 95% CI: 1.56, 7.51; *n *= 4; Heterogeneity: Chi² *P* = 0.06; I² = 60%**	
**Childhood traumatic event**				
Yes	OR 2.15; 95% CI: 0.52, 8.89; n = 1			
Pre-eclampsia	**OR 2.10; 95% CI: 1.37, 3.20; *n *= 1**			
Diabetes	OR 1.41; 95% CI: 0.95, 2.09; *n *= 1			
Congenital anomaly	OR 0.97; 95% CI: 0.59, 1.60; *n *= 1			
**History of Abortion/Miscarriage/Intrauterine Death**				
Yes	OR 1.30; 95% CI: 0.83, 2.06; *n *= 3; Heterogeneity: Chi² *P* = 0.11; I² = 55%			
No	**OR 0.19; 95% CI: 0.04, 0.98; *n *= 3; Heterogeneity: Chi² *P* < 0.00001; I² = 96%**			
No	**OR 0.15; 95% CI: 0.07, 0.29; *n *= 1**			
**Pregnancy-induced hypertension**		**OR 3.05; 95% CI: 1.38, 6.73; *n *= 1**		
**Mother blamed for child's disability**		**OR 3.48; 95% CI: 1.80, 6.71; *n *= 1**		

Note: The bold values show the significant results.

### Prevalence and risk factors of maternal antenatal depression

A total of 35 studies (Ali et al., 2012; Asad et al., 2010; Ayyub et al., 2018; Gul et al., 2017; Habiba et al., 2020; Hamid et al., 2008; Hamirani et al., 2006; Humayun et al., 2013; Imran & Haider, 2010; Irum et al., 2022; Ishtiaque et al., 2020; Jabbar et al., 2022; Jamal et al., 2018; Karmaliani et al., 2009; Kazi et al., 2006; Khan et al., 2020; Khan et al., 2021; Khanam et al., 2022; Maqbool et al., 2022; Maselko et al., 2019; Mir et al., 2012; Niaz et al., 2004; Premji et al., 2020; Rabia et al., 2017; Rahman et al., 2003 Rahman & Creed, 2007;; Sabir et al., 2019; Sadaf et al., 2011; Saeed et al., 2016; Shah et al., 2011; Shaikh et al., 2011; Waqas et al., 2015; Zahidie et al., 2011; Zareen et al., 2009; Zu et al., 2016) reported on the prevalence of maternal antenatal depression with a pooled prevalence of 37% (95% CI: 30.6% to 43.6%; cases: 5234/15409; I^2^: 98.6%).

Among the risk factors reported in the included studies, there were significantly increased odds of antenatal depression with women being multiparous (OR:2.14; 95% CI:1.14–4.01; *n* = 5), using contraception (OR:1.56; 95% CI:1.07–2.29; *n* = 2), experiencing marital problems (OR:7.57; 95% CI:2.62–21.85; *n* = 1), facing issues with parents or in-laws (OR:4.08; 95% CI:2.36–7.04; *n* = 2), dealing with family hardships (OR:2.15; 95% CI:1.72–2.70; *n* = 2), encountering housing difficulties (OR:2.81; 95% CI:1.70–4.63; *n* = 1), experiencing food insecurity (OR:3.45; 95% CI:2.59–4.60; *n* = 2), the recent death of close family relative (OR:1.97; 95% CI:1.24–3.14; *n* = 1) or parent (OR:1.81; 95% CI:1.11–2.97; *n* = 1), passive smoking (OR:2.68; 95% CI:1.73–4.17; *n* = 1), drug abuse (OR:2.48; 95% CI:1.09–5.62; *n* = 2), family history of pre-eclampsia (OR:2.10; 95% CI:1.37–3.20; *n* = 1), and having any previous psychiatric illness (OR:2.57; 95% CI:1.04–6.35; *n* = 2). In contrast, planned pregnancy (OR:0.35; 95% CI:0.14–0.85; *n* = 4), no history of abortion/miscarriage (OR:0.19; 95% CI:0.04–0.98; *n* = 3), regular physical activity (OR:0.43; 95% CI:0.20–0.89; *n* = 1), women being literate (OR:0.48; 95% CI:0.38–0.62; *n* = 5), husband being literate (OR:0.55; 95% CI:0.36–0.85; *n* = 2), having upper socio-economic status (OR:0.33; 95% CI: 0.18–0.60; *n* = 1), not being separated from husband (OR:0.18; 95% CI:0.04–0.74; *n* = 2), no domestic violence (OR:0.25; 95% CI:0.07–0.95; *n* = 5), and satisfaction with life (OR:0.22; 95% CI:0.06–0.81; *n* = 1) were found to be significantly associated with lower odds of antenatal depression.

We did not find any significant association between antenatal depression and age of the women, residence, years of education, ethnicity, occupation, type of family, number of people living in the household, monthly household income, intentions of using family planning, fear of childbirth, spousal support, housing difficulties, maternal anthropometrics, family medical history, previous adverse pregnancy outcomes. However, these findings must be interpreted with caution due to the small number of studies and high heterogeneity.

### Prevalence and risk factors of maternal postnatal depression

**Figure 3.**
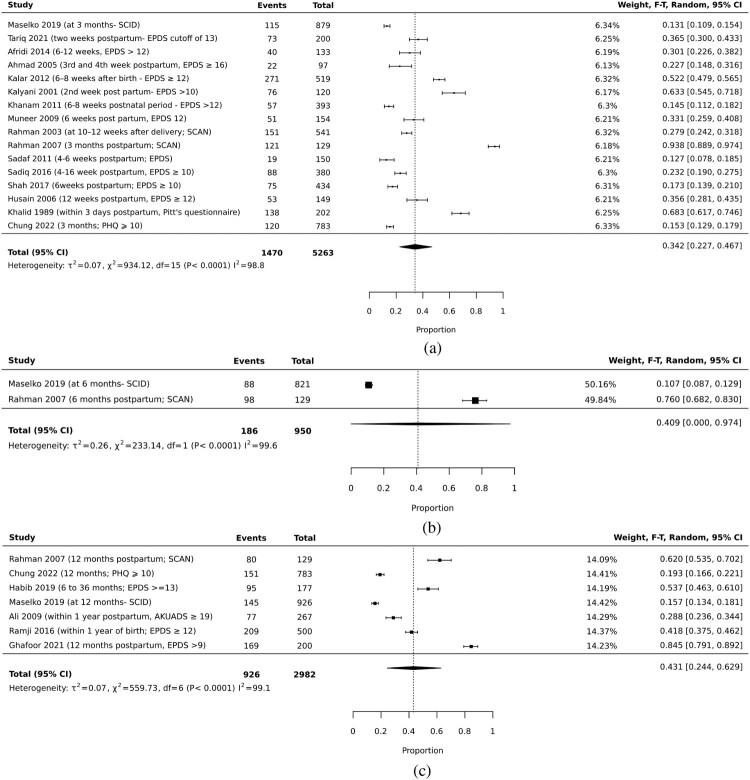
Prevalence of maternal postnatal depression. (a). Prevalence of Maternal Postnatal Depression at three months. (b). Prevalence of Maternal Postnatal Depression at six months. (c). Prevalence of Maternal Postnatal Depression at 12 months.

The prevalence of maternal postnatal depression at three months was estimated to be 34.2% (22.7% to 46.7%; 16 studies; cases: 1470/5263; I^2^: 98.8%) (Afridi et al., 2014; Ahmad & Khan, 2005; Chung et al., 2022; Husain et al., 2006; Kalar et al., 2012; Kalyani, 2001; Khalid, 1989; Khanam et al., 2011; Maselko et al., 2019; Muneer et al., 2009; Rahman et al., 2003 Rahman & Creed, 2007;; Sadaf et al., 2011; Sadiq et al., 2016; Shah & Lonergan, 2017; Tariq et al., 2021), at six months was 40.9% (0% to 97.4%; 2 studies; cases: 186/950; I^2^: 99.6%; Maselko et al., 2019; Rahman & Creed, 2007) and at 12 months was 43.1% (24.4% to 62.9%; 7 studies; cases: 926/2982; I^2^: 99.1%) (Figure 3) (Ali et al., 2009; Chung et al., 2022; Ghafoor et al., 2021; Habib & Ali, 2019; Maselko et al., 2019; Rahman & Creed, 2007; Ramji et al., 2016).

Among the risk factors reported in the included studies, there were significantly increased odds of maternal postnatal depression with being nulliparous (OR:5.30; 95% CI:1.31–21.47; *n* = 1), not receiving antenatal care (OR:3.09; 95% CI:1.09–8.82; *n* = 1), experiencing pregnancy-induced hypertension (OR:3.05; 95% CI:1.38–6.73; *n* = 1), having previous psychiatric illness (OR:7.14; 95% CI:3.54–14.37; *n* = 1), having a husband/family head who is illiterate (OR:2.02; 95% CI:1.24–3.29; *n* = 1), having a husband who is unemployed (OR:2.17; 95% CI:1.19–3.97; *n* = 1), unhappy or having problems with parents or in-laws (OR:1.62; 95% CI:1.01–2.61; n = 2), and being blamed for child disability (OR:3.48; 95% CI:1.80–6.71; *n* = 1). While urban residence (OR:0.48; 95% CI:0.24–0.97; *n* = 1), being financially independent (OR:0.45; 95% CI:0.31–0.67; *n* = 1), having no family hardships (OR:0.30; 95% CI:0.16–0.58; *n* = 1), having no problems with parents/in-laws (OR:0.52; 95% CI:0.28–0.95; *n* = 1), receiving antenatal care (OR:0.32; 95% CI:0.11–0.92; n = 1), and having help with daily child care (OR:0.38; 95% CI:0.27–0.54; *n* = 2) were found to be significantly associated with lower odds of postnatal depression.

We did not find any significant association between postnatal maternal depression and maternal age, education, ethnicity, occupation, type of family, monthly household income, nature of pregnancy, type of delivery, obstetric complications, spousal support, marital problems, domestic violence, having one or more female child, medical complications, housing difficulties, and loss of parents. However, these findings must be interpreted with caution due to the small number of studies and high heterogeneity.

### Prevalence and risk factors of paternal postnatal depression

Two (Atif et al., 2022; Noorullah et al., 2020) of the included studies also reported paternal postnatal depression with a pooled prevalence of 40.5% (14.9% to 69%; cases: 46/141; I^2^: 83.8%; Annex 7). Among the risk factors, paternal age of < 30 years (OR:11.25; 95% CI:2.13–59.38; *n* = 1), being unemployed (OR:12.67; 95% CI:1.18–136.44; *n* = 1), having financial hardships (OR:8.70; 95% CI:1.96–38.65; *n* = 1), having sleep disturbances (OR:11.63; 95% CI:2.54–53.17; *n* = 1), having spousal sleep disturbances (OR:5.36; 95% CI:1.24–23.10; *n* = 1) and having a depressed spouse (OR:11.25; 95% CI:2.13–59.38; *n* = 1) significantly increased the odds of paternal postnatal depression.

### Prevalence and risk factors of maternal perinatal depression

**Figure 4.**
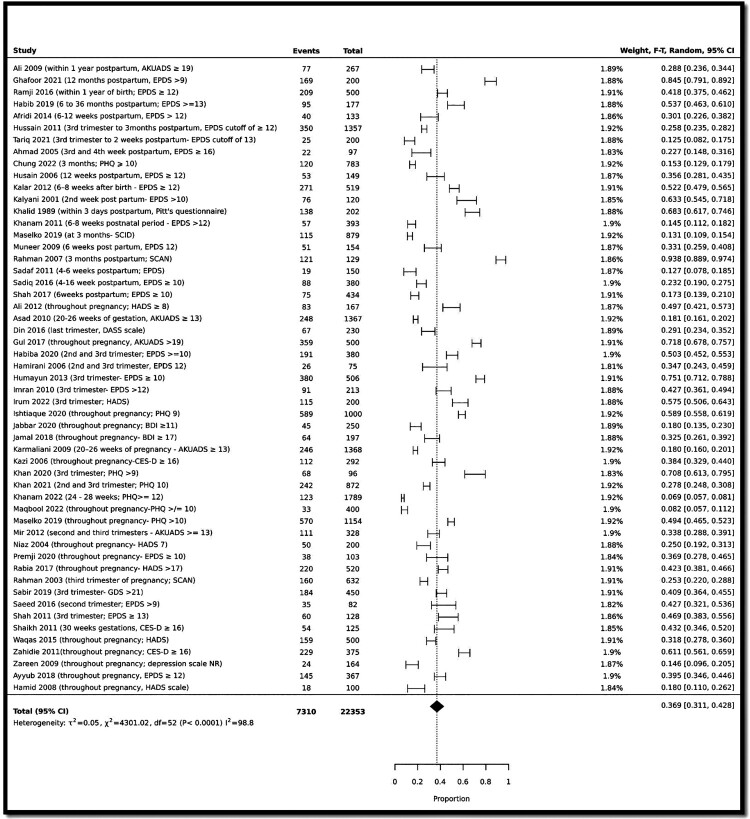
Prevalence of perinatal depression.

We pooled the prevalence of antenatal, perinatal, and postnatal depression to estimate the pooled prevalence of perinatal depression. A total of 52 studies (Afridi et al., 2014; Ahmad & Khan, 2005; Ali et al., 2009; Ali et al., 2012; Asad et al., 2010; Ayyub et al., 2018; Chung et al., 2022; Ghafoor et al., 2021; Gul et al., 2017; Habib & Ali, 2019; Habiba et al., 2020; Hamid et al., 2008; Hamirani et al., 2006; Humayun et al., 2013; Husain et al., 2006; Husain et al., 2011; Imran & Haider, 2010; Irum et al., 2022; Ishtiaque et al., 2020; Jabbar et al., 2022; Jamal et al., 2018; Kalar et al., 2012; Kalyani, 2001; Karmaliani et al., 2009; Kazi et al., 2006; Khalid, 1989; Khan et al., 2020; Khan et al., 2021; Khanam et al., 2011; Khanam et al., 2022; Maqbool et al., 2022; Maselko et al., 2019; Mir et al., 2012; Muneer et al., 2009; Niaz et al., 2004; Premji et al., 2020; Rabia et al., 2017; Rahman & Creed, 2007; Rahman et al., 2003; Ramji et al., 2016; Sabir et al., 2019; Sadaf et al., 2011; Sadiq et al., 2016; Saeed et al., 2016; Shah & Lonergan, 2017; Shah et al., 2011; Shaikh et al., 2011; Tariq et al., 2021; Zu et al., 2016; Waqas et al., 2015; Zahidie et al., 2011; Zareen et al., 2009) reported maternal perinatal depression with a pooled prevalence of 36.9% (31.1% to 42.8%; cases: 7310/22353; I^2^: 98.8%) (See Figure 4). Of 52 studies, only two studies (Husain et al., 2011; Tariq et al., 2021) specifically reported on perinatal depression.

Among the risk factors reported in included studies, there were significantly increased odds of perinatal depression with maternal illiteracy (OR:1.56; 95% CI:1.04–2.34; *n* = 11), husband/ family head illiteracy (OR:1.83; 95% CI:1.25–2.67; *n* = 3), husband unemployment (OR:2.34; 95% CI:1.51–3.63; *n* = 2), lower socio-economic status (OR:2.67; 95% CI:1.52–4.67; *n* = 2), experiencing family hardships (OR:2.20; 95% CI:1.66–2.90; *n* = 4), use of contraception (OR:1.52; 95% CI:1.08–2.15; *n* = 3), marital problems (OR:5.31; 95% CI:1.21–23.31; *n* = 3), issues with parents/in-laws (OR:2.37; 95% CI:1.32–4.27; *n* = 4), loss of parents (OR:1.89; 95% CI:1.22–2.95; *n* = 2), experiencing bereavement or illness (OR:1.49; 95% CI:1.03–2.13; *n* = 2), and having a history of previous psychiatric illness (OR:3.42; 95% CI:1.56–7.51; *n* = 4). In contrast, urban residence (OR:0.52; 95% CI:0.30–0.90; *n* = 2) and being nulliparous (OR:0.60; 95% CI:0.38–0.95; *n* = 6) were found to be significantly associated with lower odds of antenatal depression.

We did not find any significant association between perinatal depression and maternal age, occupation, nature of pregnancy, obstetric complications, type of family, monthly income, spousal support, and domestic violence. However, these findings must be interpreted with caution due to the small number of studies and high heterogeneity.

## Discussion

The present study aimed to review the prevalence and risk factors associated with perinatal depression, encompassing both antenatal and postnatal depression, among mothers and fathers in Pakistan. The review included data from 61 studies of 23,838 women and provided a comprehensive overview of the prevalence and risk factors associated with this significant public health issue. Our findings revealed a pooled prevalence of 37% for maternal antenatal depression and varying prevalence rates for maternal postnatal depression at different time points (34.2% at three months, 40.9% at six months, and 43.1% at 12 months). Additionally, we identified a pooled prevalence of 40.5% for paternal postnatal depression. The review identified numerous risk factors significantly associated with increased odds of maternal perinatal depression, including being multiparous, failure of contraceptives, insufficient antenatal care, pregnancy-induced hypertension, previous psychiatric illness, passive smoking, drug abuse, low socio-economic status, marital problems, family hardships, recent death of close family relative or parent, housing difficulties, food insecurity, head of family/husband's illiteracy, husband's unemployment, and being blamed for child disability. Paternal postnatal depression was significantly associated with paternal age below 30, unemployment, financial difficulties, and sleep disturbances in both partners and a depressed spouse. It is important to note that previous history of psychiatric illness has been a consistent contributor to antenatal, postnatal, and overall perinatal depression, which could be correlated with other contributing risk factors such as illiteracy, financial issues, or loss of close ones. Moreover, certain risk factors during the natal period showed varied associations with different types of maternal depression. Previous history of c-sections, being nulliparous, and being in the second trimester of pregnancy were found to be significantly associated with a decrease in maternal antenatal depression, while being multiparous was found to be significantly associated with an increase in maternal antenatal depression. Being nulliparous was also found to be associated with a decrease in maternal perinatal depression. However, being nulliparous was found to be significantly associated with an increase in maternal postnatal depression. Receiving no antenatal care was another risk factor significantly associated with an increased risk of maternal postnatal depression. These findings highlight the important associations of risk factors for maternal depression across different stages of pregnancy and postpartum periods.

A systematic review assessing the burden of postnatal depression in LMICs reported that one in five postnatal women were depressed in LMICs (Dadi et al., 2020). Another systematic review on antenatal depression reported a prevalence of 24.3% among South Asian women and an even higher prevalence of 32.2% among Pakistani women (Mahendran et al., 2019). These findings are consistent with the findings of the previous review by Atif et al. (2021), reporting a 37% prevalence of antenatal depression, a prevalence of 29.5% for postnatal depression and 37% for perinatal depression among Pakistani women. As compared to the previously published review our review delved further into postnatal depression by exploring its occurrence at different time points, revealing rates of 34.2% at three months, 40.9% at six months, and 43.1% at 12 months after childbirth. Thus, highlighting an increasing trend of mental health deterioration among Pakistani women. A recent review on the social determinants of antenatal depression and anxiety in South Asian women found that factors like the quality of relationships with husbands and in-laws, social support, and the gender of the child played significant roles in increasing the risk of antenatal depression (Insan et al., 2022). Consistently, our review also identified a significant association between antenatal depression and issues related to marital problems and family challenges.

Our review identified a significant gap in research reporting on only two studies assessing paternal depression. This was supported by a correspondence by Khabir in 2022 that discussed the underrepresentation of postpartum depression among males (Khabir et al., 2022). The article shed light on contributing factors to depression among men, including unemployment, number of children, financial stress, and cultural taboos surrounding seeking help for mental health issues. Our review highlighted similar factors like unemployment, lower socio-economic status, family hardships, and other risk factors associated with paternal postpartum depression, emphasizing the importance of addressing this often-overlooked aspect of perinatal mental health. A recent review assessing the prevalence of perinatal mood disorders in both mothers and fathers (parental dyad) reported that up to 3.18% of parental dyads experienced perinatal depression, and prevalence was higher in the late postnatal period (3–12 months) (Smythe et al., 2022). The review also concluded that there was insufficient data on parental perinatal anxiety to draw any conclusions (Smythe et al., 2022).

Our review has several strengths and limitations. One notable strength of this review is its comprehensiveness, as it encompasses a wide range of studies conducted over a significant period, thus providing a thorough understanding of the landscape of perinatal depression in Pakistan. Additionally, the inclusion of paternal postnatal depression adds to the completeness of the review, acknowledging the importance of mental health in fathers during this critical life transition, i.e. parenthood. Moreover, using standardised quality assessment tools enhanced the reliability of the study findings. However, there are some limitations to acknowledge. Firstly, we only included peer-reviewed and published studies in our review and did not include grey literature which may lead to publication bias. Secondly, most of the included studies were judged to be of low quality due to concerns related to sample size, study duration, adjustment of confounding variables, and risk factor assessment over time. These factors may affect the reliability and generalizability of the study findings. The short duration of some studies may not capture long-term effects or associations that develop over time, while small sample sizes and improper adjustments for confounders further limit the robustness of the conclusions. This limitation underscores the need for caution when interpreting the study findings and highlighting the necessity for more rigorous research in this field. Additionally, high heterogeneity in several findings also challenges the generalisability of results, highlighting the need for further exploration of potential sources of variation. Finally, our review finding on the high prevalence of PPD, especially at 6 and 12 months, require further evaluation as this variability could be attributable to various causes, including cultural differences, differences in study time, PPD assessment method, the cut-off point for EDPS, sample size, and study methodology.

Our findings have important implications for policy and research. The high prevalence rates of perinatal depression in Pakistan underscore the necessity for targeted healthcare interventions and support systems. Moreover, the COVID-19 epidemic has led to an exacerbation of psychological problems in high-risk population groups that include pregnant women and may have potentially accelerated an existing trend of increasing prevalence of postnatal depression, especially in resource-limited settings (Harrison et al., 2023; Safi-Keykaleh et al., 2022). Policies and healthcare programs should prioritise the early identification and management of perinatal depression, considering the associated risk factors identified in this study. Universal screening might be beneficial for high-risk parents, especially in LMIC settings, for early identification and timely management. A recent meta-analysis collating evidence on screening programmes for perinatal depression and anxiety found a positive impact on both depression and anxiety symptoms (Waqas et al., 2022). However, this would require valid and reliable assessment tools and future studies examining its cost-effectiveness (Garthus-Niegel et al., 2022). Furthermore, the findings emphasise the need for family-centred care models that encompass the mental health and well-being of both mothers and fathers during the perinatal period. From a research perspective, this study highlights the gaps and limitations in the existing literature. Future research should focus on improving study quality, exploring the sources of heterogeneity, and addressing underrepresented areas such as paternal mental health. Moreover, it is important to conduct longitudinal studies to assess the risk factors over different time points, including antenatal, perinatal, and post-natal periods. This approach is essential for a more nuanced understanding of perinatal depression.

## Conclusion

In conclusion, the findings of this study contribute to the growing body of evidence on perinatal depression in Pakistan and emphasise the importance of addressing mental health challenges during the perinatal period. Effective policies and further research in this area can improve healthcare outcomes and better support for families during this critical phase of life.

## Supplementary Material

Supplementary file.pdf
