# Supplementary material for: Prevalence and risk factors of perinatal depression among mothers and fathers in Pakistan: a systematic review and meta-analysis
Source: Health Psychol Behav Med. 2024 Aug 9;12(1):2383468. doi: 10.1080/21642850.2024.2383468 (PMC11318492; doi:10.1080/21642850.2024.2383468)
Supplement: Supplementary file.pdf [file RHPB_A_2383468_SM1951.pdf]

Annexure

Contents

Annexe 1: PRISMA Checklist 2

Annex 2: Search Strategies 4

Annexe 3: Characteristics of Included Studies 6

Annexe 4: Quality Assessment of Included Studies 19

4a: Quality Assessment of Cohort and Cross-sectional Studies 19

4b: Quality Assessment of Case-control Studies 21

Annexe 5: Risk Factors Associated with Maternal Perinatal Depression 22

Annexe 6: Risk Factors Associated with Paternal Postnatal Depression 34

Annexe 7: Forest on the Prevalence of Paternal Postnatal Depression 35

References 36

Annexe 1: PRISMA Checklist

Section and Topic	Item #	Checklist item	Location where item is reported
**TITLE**			
Title	1	Identify the report as a systematic review.	1
**ABSTRACT**			
Abstract	2	See the PRISMA 2020 for Abstracts checklist.	2
**INTRODUCTION**			
Rationale	3	Describe the rationale for the review in the context of existing knowledge.	4-5
Objectives	4	Provide an explicit statement of the objective(s) or question(s) the review addresses.	5
**METHODS**			
Eligibility criteria	5	Specify the inclusion and exclusion criteria for the review and how studies were grouped for the syntheses.	5-6
Information sources	6	Specify all databases, registers, websites, organisations, reference lists and other sources searched or consulted to identify studies. Specify the date when each source was last searched or consulted.	6
Search strategy	7	Present the full search strategies for all databases, registers and websites, including any filters and limits used.	Annex 2
Selection process	8	Specify the methods used to decide whether a study met the inclusion criteria of the review, including how many reviewers screened each record and each report retrieved, whether they worked independently, and if applicable, details of automation tools used in the process.	6
Data collection process	9	Specify the methods used to collect data from reports, including how many reviewers collected data from each report, whether they worked independently, any processes for obtaining or confirming data from study investigators, and if applicable, details of automation tools used in the process.	6
Data items	10a	List and define all outcomes for which data were sought. Specify whether all results that were compatible with each outcome domain in each study were sought (e.g. for all measures, time points, analyses), and if not, the methods used to decide which results to collect.	6-7
	10b	List and define all other variables for which data were sought (e.g. participant and intervention characteristics, funding sources). Describe any assumptions made about any missing or unclear information.	4-5
Study risk of bias assessment	11	Specify the methods used to assess risk of bias in the included studies, including details of the tool(s) used, how many reviewers assessed each study and whether they worked independently, and if applicable, details of automation tools used in the process.	6-7
Effect measures	12	Specify for each outcome the effect measure(s) (e.g. risk ratio, mean difference) used in the synthesis or presentation of results.	7
Synthesis methods	13a	Describe the processes used to decide which studies were eligible for each synthesis (e.g. tabulating the study intervention characteristics and comparing against the planned groups for each synthesis (item #5)).	7
	13b	Describe any methods required to prepare the data for presentation or synthesis, such as handling of missing summary statistics, or data conversions.	7
	13c	Describe any methods used to tabulate or visually display the results of individual studies and syntheses.	7
	13d	Describe any methods used to synthesize results and provide a rationale for the choice(s). If meta-analysis was performed, describe the model(s), method(s) to identify the presence and extent of statistical heterogeneity, and software package(s) used.	7
	13e	Describe any methods used to explore possible causes of heterogeneity among study results (e.g. subgroup analysis, meta-regression).	7
	13f	Describe any sensitivity analyses conducted to assess robustness of the synthesized results.	NA
Reporting bias	14	Describe any methods used to assess risk of bias due to missing results in a	7

Section and Topic	Item #	Checklist item	Location where item is reported
assessment		synthesis (arising from reporting biases).	
Certainty assessment	15	Describe any methods used to assess certainty (or confidence) in the body of evidence for an outcome.	NA
**RESULTS**			
Study selection	16a	Describe the results of the search and selection process, from the number of records identified in the search to the number of studies included in the review, ideally using a flow diagram.	7-8
	16b	Cite studies that might appear to meet the inclusion criteria, but which were excluded, and explain why they were excluded.	7-8
Study characteristics	17	Cite each included study and present its characteristics.	Annex 3
Risk of bias in studies	18	Present assessments of risk of bias for each included study.	8-9 and Annex 4
Results of individual studies	19	For all outcomes, present, for each study: (a) summary statistics for each group (where appropriate) and (b) an effect estimate and its precision (e.g. confidence/credible interval), ideally using structured tables or plots.	7-12
Results of syntheses	20a	For each synthesis, briefly summarise the characteristics and risk of bias among contributing studies.	Annex 4
	20b	Present results of all statistical syntheses conducted. If meta-analysis was done, present for each the summary estimate and its precision (e.g. confidence/credible interval) and measures of statistical heterogeneity. If comparing groups, describe the direction of the effect.	7-12, Annex 5 and 6
	20c	Present results of all investigations of possible causes of heterogeneity among study results.	7-12
	20d	Present results of all sensitivity analyses conducted to assess the robustness of the synthesized results.	NA
Reporting biases	21	Present assessments of risk of bias due to missing results (arising from reporting biases) for each synthesis assessed.	Annex 4
Certainty of evidence	22	Present assessments of certainty (or confidence) in the body of evidence for each outcome assessed.	NA
**DISCUSSION**			
Discussion	23a	Provide a general interpretation of the results in the context of other evidence.	12
	23b	Discuss any limitations of the evidence included in the review.	14
	23c	Discuss any limitations of the review processes used.	14
	23d	Discuss implications of the results for practice, policy, and future research.	15
**OTHER INFORMATION**			
Registration and protocol	24a	Provide registration information for the review, including register name and registration number, or state that the review was not registered.	5
	24b	Indicate where the review protocol can be accessed, or state that a protocol was not prepared.	5
	24c	Describe and explain any amendments to information provided at registration or in the protocol.	NA
Support	25	Describe sources of financial or non-financial support for the review, and the role of the funders or sponsors in the review.	16
Competing interests	26	Declare any competing interests of review authors.	16
Availability of data, code and other materials	27	Report which of the following are publicly available and where they can be found: template data collection forms; data extracted from included studies; data used for all analyses; analytic code; any other materials used in the review.	16

From: Page MJ, McKenzie JE, Bossuyt PM, Boutron I, Hoffmann TC, Mulrow CD, et al. The PRISMA 2020 statement: an updated guideline for reporting systematic reviews. BMJ 2021;372:n71. doi: 10.1136/bmj.n71

For more information, visit: http://www.prisma-statement.org/

Annex 2: Search Strategies

Medline:

1 exp Depression, Postpartum/ 7408

2 (antenatal or perinatal or peri-natal or prenatal or pre-natal or postnatal or post-natal or postpartum or post-partum or mother* or father* or puerperal).ti,ab. 605342

3 1 or 2 605817

4 (depression or major depressive disorder).ti,ab. 423705

5 exp Pakistan/ 22593

6 3 and 4 and 5 111

7 limit 6 to (humans and yr="2019 -Current") 43

Embase

1 exp postnatal depression/ 7211

2 (antenatal or perinatal or peri-natal or prenatal or pre-natal or postnatal or post-natal or postpartum or post-partum or mother* or father* or puerperal).ti,ab. 785977

3 1 or 2 786985

4 (depression or major depressive disorder).ti,ab. 591202

5 exp Pakistan/ 37133

6 3 and 4 and 5 187

7 limit 6 to (human and yr="2019 -Current") 88

PsychInfo

1 exp Postpartum Depression/ 5946

2 (antenatal or perinatal or peri-natal or prenatal or pre-natal or postnatal or post-natal or postpartum or post-partum or mother* or father* or puerperal).ti,ab. 201894

3 1 or 2 202006

4 (depression or major depressive disorder).ti,ab. 289573

5 Pakistan.mp. 4059

6 3 and 4 and 5 88

7 limit 6 to (human and yr="2019 -Current") 28

CINAHL

(MH "Depression, Postpartum") OR (antenatal OR puerperal OR perinatal OR peri-natal OR prenatal OR pre-natal OR postnatal OR post-natal OR postpartum OR post-partum OR mother* OR father*)

AND

(depression OR "major depressive disorder")

AND

(MH "Pakistan")

Hits: 72

Limits 2019 to current

Hit: 19

Annexe 3: Characteristics of Included Studies

Author, Year	Study Design	Participants (N)	Geographical Location	Urban/Rural	Hospital/ Community	Instrument to measure depression	Cutoff	Maternal (antenatal -postnatal)/paternal depression	Risk Factors
1. Afridi 2014[1]	Cross-sectional study	Women, 6-12 weeks postpartum, both primipara and multipara with mean age of 26.23 ± 2.64 years (n= 133)	Hayatabad Peshawar	Urban	Hospital	Edinburgh Postnatal Depression Scale (EPDS)	> 12	Postnatal depression	Age, parity, mode of delivery
2. Ahmad 2005[2]	Cross-sectional study	Post-natal women, 3rd and 4th week postpartum (n= 97)	Lahore	Urban	Hospital	EPDS	≥ 16	Postnatal depression	Nature of pregnancy, gender of baby, income level, type of delivery
3. Ali 2009[3]	Cohort study	Pregnant women (n= 267)	Karachi	Peri-urban	Community	The Aga Khan University Anxiety and Depression Scale (AKUADS)	≥ 19	Postnatal depression (within 1 year postpartum)	Study area, maternal age, employment, migration status, past history of child death, unplanned current pregnancy, domestic violence, complication during pregnancy, any difficulty soon after birth for the baby, any difficulty in breast feeding at birth
4. Ali 2012[4]	Cross-sectional study	Pregnant women (n= 167)	Karachi	Urban	Hospital	Hospital Anxiety Depression Scale (HADS)	≥ 8	Antenatal depression	Age, ethnicity, education, employment status, people living in the house, total pregnancies, total live births, adverse pregnancy outcomes, willingness of pregnancy, use of family planning methods, self-treated or any family member for psychiatric disorder, satisfied with life, household environment, help seeking behaviour, household decision maker, domestic violence
5. Asad 2010[5]	Cohort	Pregnant women, 20-26 weeks of gestation (n= 1369)	Hyderabad	Urban	Community	AKUADS	≥ 13	Antenatal depression	Domestic abuse and education

Author, Year	Study Design	Participants (N)	Geographical Location	Urban/Rural	Hospital/ Community	Instrument to measure depression	Cutoff	Maternal (antenatal -postnatal)/paternal depression	Risk Factors
6. Atif 2022[6]	Cross-sectional study	Husbands of wives attending antenatal clinic (n= 73)	Karachi	Urban	Hospital	EPDS and the Pittsburgh Sleep Quality Index (PSQI)	EPDS: >10<br><br>PSQI: score of 5 indicates poor sleep quality	Paternal postnatal depression	Age (<30), employment, financial hardship, number of children, sleep disturbance, spouse screened positive for depression, satisfaction with marital relationship
7. Ayyub 2018[7]	Cross-sectional study	Pregnant women (throughout pregnancy) aged 18 to 45 years (n= 367)	Lahore	Urban	Community	EPDS	≥ 12	Antenatal depression	Maternal age, unwanted pregnancy, death of close relative (within 1 year), intimate partner violence, food insecurity
8. Brown 2021[8]	Better Lives Study – Foundational Research (EBLS-FR) birth cohort study	Women in third trimester of pregnancy (n = 134)	NR	NR	Local health centers	Patient Health Questionnaire (PHQ-9)	PHQ: Scores range from 0 to 27, with higher scores indicative of more severe depressive symptoms	Antenatal depression	Adverse Childhood Experiences
9. Chung 2022[9]	Cohort with an embedded cluster-randomized trial	Pregnant women in the third trimester of pregnancy (18 years or older)	Rawalpindi	Rural	Community	PHQ-9	⩾ 10	Maternal postnatal depression	Mother-in-law childcare and family conflict
10. Din 2016[10]	Cross-sectional study	Women >18 years, having gestational age of >27 weeks (last trimester) (n= 230)	Peshawar	Semi-urban	Hospital	Depression Anxiety Stress Scale (DASS-42)	NR	Antenatal depression	Maternal age, monthly income, parity, home status, family type, husband support, monthly income, family size, problems with the in laws, history of depression, life events, home ownership, lack of confidence, gravidity, youth children in family, pregnancy related concern, domestic violence
11. Ghaffar 2017[11]	Cross-sectional study	Pregnant women in their fourth pregnancy aged 31 to 40 years (n= 752)	Quetta	Urban/rural	Hospital	HADS	NR	Antenatal depression	Age, education, occupation, monthly income, locality, ethnic group, number of children

Author, Year	Study Design	Participants (N)	Geographical Location	Urban/Rural	Hospital/ Community	Instrument to measure depression	Cutoff	Maternal (antenatal -postnatal)/paternal depression	Risk Factors
12. Ghafoor 2021[12]	Cross-sectional study	Women who delivered in year 2018; mean age 28.14(±4.935) years (n= 200)	Karachi	NR	Hospital	EPDS, Body Image Dissatisfaction (BID) Questionnaire	0-8: depression less likely 9-11: depression possible 12-13: fair possibility of depression 14 and high: high possibility of depression	Maternal postpartum depression	Education, occupation, age, parity, body image dissatisfaction
13. Gul 2013[13]	Cohort	Pregnant women mean age of 28.3±6.3 years – 16 to 27 days of delivery (n= 500)	Lahore	Urban slums	Hospital	EPDS and Siddiqui Shah Depression Scale	NR	Postnatal depression	Birth of a female child, socioeconomic conditions, family history of psychiatric illness
14. Gul 2017[14]	Cross-sectional study	Pregnant women mean age of 28.3±6.3 years (throughout pregnancy) (n= 500)	Kohat	Urban	Hospital	AKUADS	>19	Antenatal depression	Stressors, stress level, marriage, alive male and female children, income, occupation
15. Habib 2019[15]	Cross-sectional study	Mothers aged at least 17 years of children with movement disorders, and had no history of psychiatric illness, chronic physical illness or learning disability (n= 171)	Peshawar	NR	Rehabilitation centre	EPDS	≥13	Prolonged Postpartum depression	Type of family, Husband is the only breadwinner, stressful Life events, social support, antenatal care received, type of delivery, pregnancy induced hypertension, baby blues, disability
16. Habiba 2020[16]	Cross-sectional study	Pregnant women, aged 18-45 years, in their 2nd and 3rd trimester (n=380)	Faisalabad	NR	Hospital	EPDS	⩾ 10	Prenatal depression	Education, education of head of family, socio-economic class, BMI, pregnancy duration, history of miscarriage, contraception, stressful life events, women autonomy/decision
17. Hamid 2008[17]	Cross-sectional study	Pregnant women (n= 100)	Lahore	Urban	Hospital	HADS	NR	Antenatal depression	NR
18. Hamirani 2006[18]	Cross-sectional study	Pregnant women in their 2nd and 3rd trimester with mean	Karachi	Urban	Hospital	EPDS	12	Antenatal depression	Age, parity, trimester

Author, Year	Study Design	Participants (N)	Geographical Location	Urban/Rural	Hospital/ Community	Instrument to measure depression	Cutoff	Maternal (antenatal -postnatal)/paternal depression	Risk Factors
		age of 26 years (n= 75)							
19. Humayun 2013[19]	Cross-sectional study	Pregnant women in their 3rd trimester aged 20-30 years (n= 506)	Lahore	Urban	Hospital	EPDS	≥ 10	Antenatal depression	Age, nature of pregnancy, fear of childbirth, miscarriage or intrauterine death, lack of support, separation from husband, domestic violence, drug abuse, previous psychiatric illness, family history of previous psychiatric illness
20. Husain 2006[20]	Cross-sectional study	Pregnant women in their last trimester (n= 149)	Rawalpindi	Rural	Community	EPDS	≥ 12	Postnatal depression	Female infant gender, older maternal age, education, number of children, income, life events, social Support using MSPSS tool
21. Husain 2011[21]	Cohort	Pregnant women in their 3rd trimester aged 19 to 50 years (n= 1357)	Karachi	Urban	Hospital	EPDS	≥ 12	Perinatal depression	Monthly income, education, work outside home, husband's education and job, husband away from home for more than 1 month, number of children, ever lost a child, separate family, aged >25 at marriage, any medication taken during pregnancy, medication use, problem with last delivery, life events, disability using BDQ tool, psychological distress during pregnancy and after childbirth using SRQ tool
22. Imran 2010[22]	Cohort	Pregnant women in their 3rd trimester aged 18-35 years (n= 213)	Lahore	Urban	Hospital	EPDS	>12	Antenatal depression	Income, family structure, problems with parents/ in-laws, quality of marital relationship, history of domestic violence, family history of psychiatric illness, pressure from family for a male child, past psychiatric history, adverse life events in a year before pregnancy,

Author, Year	Study Design	Participants (N)	Geographical Location	Urban/Rural	Hospital/ Community	Instrument to measure depression	Cutoff	Maternal (antenatal -postnatal)/paternal depression	Risk Factors
									obstetric risk factors, obstetric and neonatal outcomes
23. Irum 2022[23]	Cross-sectional study	Pregnant women (n= 200)	Abbottabad	NR	Healthcare facility	HADS	NR	Antenatal anxiety and depression	Characteristics of the home environment, family interactions, obstetrics, Urdu ethnicity, low educational attainment, high socioeconomic status, spouse working in management, in-laws' decision making of family matters, and joint family structure, be in 3rd trimester and primigravida (1st pregnancy)
24. Ishtiaque 2020[24]	Cross-sectional study	Women attending the antenatal clinics for routine follow-up visits- mean age 29.4±5.5 years (n= 1000)	Karachi	NR	Hospital	PHQ-9	0 (no depressive symptoms) to 27 (most severe depressive symptoms)<br><br>0-4 indicates no to minimal depressive symptoms; increasing score indicates an increasing severity of depression.	Antenatal depression	Number of children, age, previous history of miscarriage, previous history of stillbirth, unplanned pregnancy, family history of psychiatric illness
25. Jabbar 2022[25]	Cross-sectional study	Pregnant females of age 18 to 45 with parity <5	Lahore	NR	Hospital	BDI score	≥11	Antenatal depression	Age, gestational age, parity, BMI, occupation
26. Jamal 2018[26]	Cross-sectional study	Pregnant women (n= 197)	Lahore	Urban	Hospital	Beck's Depression Inventory (BDI)	≥ 17	Antenatal depression	Age, education, gestational age, gravidity, parity, history of abortion/miscarriage, fear of childbirth, history of c-section, family type, household income,

Author, Year	Study Design	Participants (N)	Geographical Location	Urban/Rural	Hospital/ Community	Instrument to measure depression	Cutoff	Maternal (antenatal -postnatal)/paternal depression	Risk Factors
									nature of pregnancy, childhood traumatic events, separation from husband and stressful life events
27. Kalar 2012[27]	Cross-sectional study	6–8 weeks after birth (n= 519)	Karachi	Urban	Community	EPDS	≥ 12	Postnatal depression	Age, antenatal and post-natal risk factors, education, occupation
28. Kalyani 2001[28]	Cross-sectional study	Women in full term pregnancy (n= 120)	Rawalpindi	Urban	Hospital	EPDS	>10	Postnatal depression (2nd week post partum)	Unwanted pregnancy, being primipara, living in joint family system, relationship and marital difficulties, early loss of mother.
29. Karmaliani 2009[29]	Cross-sectional study	Pregnant women in their 20–26 weeks of pregnancy (n= 1368)	Hyderabad	Urban	Community	AKUADS	≥ 13	Antenatal depression	Age, education, employment, husband's employment, property index, number of previous pregnancies, wanted this pregnancy, domestic violence within six months of this pregnancy
30. Kazi 2006[30]	Cross-sectional study	Pregnant women (n= 292)	Karachi	Urban	Hospital	Center for Epidemiological Studies - Depression (CES-D) scale	≥ 16	Antenatal depression	Social relation and conditions, pregnancy-related concerns, husband, in-laws, children, illness, economy, life events, household work, social problems, unborn baby, pregnancy symptoms, concern for changes during pregnancy, dependency due to pregnancy
31. Khalid 1989[31]	Case-control	Mothers with normal delivery aged 18-34 years (n= 202)	Lahore	Urban	Hospital	Pitt's questionnaire for Puerperal depression	NR	Postnatal depression	Maternity blues
32. Khan 2020[32]	Cross-sectional study	Last trimester pregnant women with mean age 24.56±4.24 years (n= 96)	Abbottabad	Rural and urban	Hospital	PHQ-9	0-9 = normal	Maternal antenatal depression	Age, height, education, place, Pregnancy-related low back pain (PLBP), total number of pregnancies, duration of pregnancies, number of previous c-

Author, Year	Study Design	Participants (N)	Geographical Location	Urban/Rural	Hospital/ Community	Instrument to measure depression	Cutoff	Maternal (antenatal -postnatal)/paternal depression	Risk Factors
									section, normal vaginal deliveries
33. Khan 2021[33]	Cross-sectional study	Women in the second and third trimester of pregnancy, mean age of 26.9 (±4.80) years (n= 500)	Rawalpindi	Rural	Community	PHQ-9	10	Prenatal depression	Maternal social support Index (MSSI), age, BMI, education, infant death, child mortality, miscarriage, number of living children, duration of pregnancy, health in last 30 days, delivery place, plan to use modern contraceptives, number of pregnancies, employment, husband income, total income, women empowerment status, wealth index, have money for basic needs, money for food, family debt, family structure, husband away from home in last 6 months, life satisfaction, expectation for life satisfaction in next four years
34. Khanam 2011[34]	Cross-sectional study	Women in the 6-8 weeks postnatal period (n= 393)	Karachi	Sub-urban	Hospital	EPDS	>12	Postnatal depression	Parity, education, socio-economic status, history of psychotrophic drug use
35. Khanam 2022 [35]	Cohort study	Pregnant women (N= 1,789 from Pakistan)	Karachi (Multi-country study)	Peri-urban	Community	PHQ-9	None or mild depression (PHQ-9 < 12) and moderate to moderately severe depression (PHQ-9 ≥ 12)	Maternal antenatal depression	Age, parity, mother's education, BMI, any tobacco use, history of diabetes, hypertension, stillbirth and miscarriage, husband education, wealth quintiles
36. LeMasters 2020[36]	Cohort study from Bachpan study, a cluster randomized perinatal	Pregnant women (n= 823)	North Punjab	Rural	NR	PHQ-9	10	Postpartum depression	Chilla*, duration of chilla, chores, female support, diet, satisfaction

Author, Year	Study Design	Participants (N)	Geographical Location	Urban/Rural	Hospital/ Community	Instrument to measure depression	Cutoff	Maternal (antenatal -postnatal)/paternal depression	Risk Factors
	depression trial nested in a longitudinal birth cohort								
37. Maqbool 2022[37]	Cross-sectional study	Pregnant females aged 18-49 years (n= 400)	Lahore	Rural and urban	Hospital	PHQ-9 and coronavirus anxiety scale (CAS)	Minimal to mild depression (PHQ-9 score < 10) and moderate to severe depression (≥ 10)<br><br>CAS cumulative score ≥ 9 denoted probabilities of having dysfunctional anxiety	Antenatal depression	Number of pregnancies, age, duration of pregnancy, regular physical activity, spouse support, perceived vulnerability of COVID-19, concerns about getting prenatal care, financial problems during pandemic, remained in isolation during this pregnancy, fear that baby might get infected after birth
38. a. Maselko 2018[38]	Cohort study	Pregnant women aged 18-45 years (n= 1154)	NR	Rural	Community	Patient health questionnaire	>10	Antenatal depression	Age, number of living children, food security, family debt, women education, husband education, assets
38. b. Maselko 2019[39]	Secondary data analysis	Pregnant women with mean age 26.6 (4.40) years (n= 996)	Rawalpindi	Rural	Community	Structured Clinical Interview for DSM disorders (SCID)	NR	Maternal post-partum depression	Father involvement
39. Masood 2017[40]	Cross-sectional study	Women with first pregnancy within the age range of 19-31years (n= 80)	Lahore	Urban	Hospital	Zung Self-Rating Depression Scale	NR	NR	Age, duration of marriage, education, age of husband, education of husband
40. Mir 2012[41]	Cross-sectional study	Pregnant women in their second and third trimesters (n= 328)	Chitral	Rural	Hospital	AKUADS	> = 13	Antenatal depression	Age, education, employment, family monthly income, family system, parity, present pregnancy status, presence of husband, marital

Author, Year	Study Design	Participants (N)	Geographical Location	Urban/Rural	Hospital/ Community	Instrument to measure depression	Cutoff	Maternal (antenatal -postnatal)/paternal depression	Risk Factors
									satisfaction, loss of parents, loss of in-laws, history of abuse, substance use
41. Muneer 2009[42]	Cross-sectional study	Women attending postnatal clinic (n= 154)	Rawalpindi	Urban	Hospital	EPDS Hamilton rating scale for depression	12 for EPDS<br><br>≥ 8 for Hamilton rating score	Postnatal depression	Age, monthly income, years married, education, occupation, number of children, place of residence, living conditions, method of delivery, obstetric complications, medical complications
42. Niaz 2004[43]	Cross-sectional study	Pregnant women throughout pregnancy (n= 200)	Lahore	Urban	Hospital	HADS	7	Antenatal depression	Age, education, gravida, duration of pregnancy, number of children, history of loss of parent during childhood, history of psychiatric illness during previous pregnancies, family history of psychiatric illness
43. Noorullah 2020[44]	Cross sectional survey	Participants were aged between 20-50 years (n= 120)	Karachi	Urban	Hospital	EPDS	Mothers: >13<br>Fathers: >10	Postnatal depression	Gender of baby, marriage, mother experienced depression during pregnancy and after delivery, birth defects in newborn, previous history of mental illness in father or in mother, family setup, newborn's mother health, pregnancy complications
44. Premji 2020[45]	Cohort study	Pregnant women aged between 18–43 years of age (n= 282)	Hyderabad and Karachi	NR	Hospital	Pregnancy-related anxiety (PRA) scale, Edinburgh depression scale (EDS), perceived stress (PS) scale, Adverse Childhood (ACE)-International Questionnaire (IQ)	EDS >9: mild-to-severe depression, EDS ≥ 13: moderate-to-severe depression<br><br>PRA scale: woman was classified as suffering from	Anxiety and Maternal perinatal depression	Recruitment site, iron supplements, adverse childhood experiences (ACE) levels, food insecurity, number of supplements

Author, Year	Study Design	Participants (N)	Geographical Location	Urban/Rural	Hospital/ Community	Instrument to measure depression	Cutoff	Maternal (antenatal -postnatal)/paternal depression	Risk Factors
							anxiety if she answered "very true" to 3 or more of the 10 items		
45. Rabia 2017[46]	Cross-sectional study	Pregnant women- throughout pregnancy (n= 520)	Karachi	Urban	Hospital	Hamilton	> 17	Antenatal depression	Education, employment, domestic violence, unsatisfactory relationship with partner, difficult relationship with in-laws, sleep disturbance, parity, gestational age, inadequate prenatal care, unplanned pregnancy
46. Rahman 2003[47]	Cohort	Pregnant women aged 17–40 years in their third trimester of pregnancy 6 weeks before delivery and again at 10–12 weeks after delivery (n= 632)	Kahuta	Rural	Community	SCAN	NR	Antenatal and postnatal depression	Age, life events, number of children, number of girl children, able to complete 'chilla' period, daily support in childcare by at least one family member, living in extended family, infant's grandmother lives with family, education, husband education, income, poverty, financially independent, significant other made redundant, financial difficulties, housing difficulties, major arguments, relationship difficulty, serious marital problems, bereavement, major illness in family, social role change, problems with the law, lack of friend or confidant
47. Rahman 2007[48]	Cohort study	Pregnant women aged 17–40 years in their third trimester of pregnancy and 3, 6 and 12 months postnatal (n= 129)	Rawalpindi	Rural	Community	SCAN	NR	Antenatal and postnatal depression	Age, life events, number of children, number of girl children, female gender of newborn, lack of social support, nuclear family, education, husband

Author, Year	Study Design	Participants (N)	Geographical Location	Urban/Rural	Hospital/ Community	Instrument to measure depression	Cutoff	Maternal (antenatal -postnatal)/paternal depression	Risk Factors
									education, husband employment, income, poverty, financially empowered, significant other made redundant, housing difficulties, major arguments, relationship difficulty, serious marital problems, bereavement, major illness in family, social role change, problems with the law, loss of friend and lack of confidant
48. Ramji 2016[49]	Cross-sectional study	Working and non-working group of postpartum mothers within 1 year of birth with mean age 28.8 ±4.9 years (n= 500)	Karachi	Urban	Community	EPDS<br><br>Postnatal Risk Questionnaire (PNRQ) Score	≥ 12 EPDS	Postnatal depression	Working women, nature of pregnancy, family support, husband support, in laws and friends support, family structure
49. Riaz 2020[50]	Cross-sectional study	Pregnant women (n= 100)	Sargodha	NR	Healthcare centers	EPDS	≥ 10	Maternal pre and postnatal depression and anxiety	Gravida and age
50. Sabir 2019[51]	Cross-sectional study	Pregnant females in their last trimester with mean age of 27.5 ±4.3 years (n= 450)	Lahore	NR	Hospital	Goldberg's depression scale	>21	Maternal antenatal depression	Family income, passive smoker, Congenital Abnormalities in Family, Family History of Pre-eclampsia, depression and diabetes mellitus
51. Sadaf 2011[52]	Cohort	Pregnant women aged 17 – 40 years; throughout pregnancy and 4-6 weeks postpartum (n= 150)	Rawalpindi	Urban	Hospital	EPDS and Hamilton Depression Scale	NR	Maternal antenatal and postnatal depression	Age, gravida, patients with history of postnatal and antenatal depression in previous pregnancies, socio-economic status, history of psychiatric history, family history of depression
52. Sadiq 2016[53]	Cross-sectional study	Postpartum females 4-16 week postpartum (n= 380)	Rawalpindi/Islamabad	Urban	Hospital	EPDS	≥ 10	Maternal postnatal depression	Family setup, emotional reaction, baby sex, age in years, education, parity

Author, Year	Study Design	Participants (N)	Geographical Location	Urban/Rural	Hospital/ Community	Instrument to measure depression	Cutoff	Maternal (antenatal -postnatal)/paternal depression	Risk Factors
53. Saeed 2016[54]	Cohort	Pregnant women in second trimester, aged 18–49 years (n= 82)	Lahore	Urban	Hospital	EPDS	> 9	Maternal antenatal depression	Age, weight, household income, education, BMI, parity, baseline and endline maternal dietary intake, neonatal outcomes
54. Shah 2011[55]	Cross-sectional study	Pregnant women in 3rd trimester (n= 128)	Gilgit Baltistan	Rural	Community	EPDS	≥ 13	Maternal antenatal depression	Age, gestation, gravida, history of abortion, marital status, use of birth control, education, employment, income, emotional, physical and sexual abuse, health perception
55. Shah 2017[56]	Cross-sectional study	Mothers aged 18-44 years with 6 weeks postpartum (n= 434)	Kahuta and Islamabad	Urban/rural	Hospital	EPDS	≥ 10	Maternal postnatal depression	Husband's employment, family support, husband support, sex of last baby, rural/urban setting, feeding status
56. Shahid 2022[57]	Cross-sectional study	Pregnant women in their third trimester age between 19 and 35 years (n= 136)	Sargodha, Faisalabad, and Lahore	NR	Hospital	Pakistan Anxiety and Depression Questionnaire (PADQ), Relationship Assessment Scale (RAS), Multidimensional Scale of Perceived Social Support (MSPSS), Childbirth Attitudes Questionnaire (CAQ)	PADQ: A score of 6 or more shows probable anxiety or depressive disorder and a score of 6 or more on the depression scale shows probable depressive disorder.	Perinatal depression	NR
57. Shaikh 2011[58]	Cohort	Pregnant women (28 to 30 weeks gestation) (n= 125)	Karachi	Urban	Hospital	CES-D	≥ 16	Maternal antenatal depression	Stress levels and preterm birth
58. Tariq 2021[59]	Cohort study	Women in their third trimester of pregnancy with mean age of 27.1±5.08 years (n= 200)	Islamabad	NR	Hospital	EPDS	13	Perinatal and two weeks postpartum depression	Age, gestational age, parity, gravida, planned/ unplanned pregnancy, hormonal contraceptive, social class, location

Author, Year	Study Design	Participants (N)	Geographical Location	Urban/Rural	Hospital/ Community	Instrument to measure depression	Cutoff	Maternal (antenatal -postnatal)/paternal depression	Risk Factors
59. Waqas 2015[60]	Cross-sectional study	Pregnant women mean age of 27.41 years (n= 500)	Lahore	Urban/rural/ semi urban	Hospital	HADS	NR	Maternal antenatal depression	Age, episiotomy, c-section, vaginal delivery, social support, ethnicity, education, occupation, household income, harassment, abortion, duration of marriage, nature of pregnancy, number of sons and daughters, people living in the house, household decision making, menstrual history, live births, stillbirths, smoking, substance use, abortions, fight with in-laws, past psychiatric history, family psychiatric history, miscarriage, death of child, parent or husband, long illness, relationship problems, harassment, domestic violence
60. Zahidie 2011[61]	Cross-sectional study	Pregnant women throughout pregnancy (n= 375)	Dadu, Khairpur, Hyderabad	Rural	Community	CES-D	≥ 16	Maternal antenatal depression	Social relation and conditions, pregnancy related concerns, age, number of alive children, abortion, gravida, husband, in-laws, children, parents, illness, economy, life events, work relation, social environment and problems, unborn baby
61. Zareen 2009[62]	Cohort	Pregnant women (n= 164)	Karachi	Urban	Hospital	NR	NR	NR	History of abuse

*Note: Chilla is a common postpartum practice in Pakistan. It is defined as a 40-day period of confinement after childbirth in which a woman returns to her mother's home, is fed fortifying foods, is exempt from household responsibilities, stays indoors, and receives additional support.[36]

Adverse Childhood Expereinces (ACE); The Aga Khan University Anxiety and Depression Scale (AKUADS); Brief Disability Questionnaire (BDQ); Childbirth Attitudes Questionnaire (CAQ); Center for Epidemiological Studies - Depression (CES-D) scale; Depression Anxiety Stress Scale (DASS-42); Edinburgh Postnatal Depression Scale (EPDS); Hospital Anxiety and Depression Scale (HADS); International Questionnaire (IQ); Multidimensional Scale of Perceived Social Support (MSPSS); Pakistan Anxiety and Depression Questionnaire (PADQ); Personal Information Questionnaire (PIQ); Pregnancy-related anxiety (PRA) scale; Perceived stress (PS) scale, Patient Health Questionnaire (PHQ-9) Pittsburgh Sleep Quality Index (PSQI); Relationship Assessment Scale (RAS); Self-Reporting Questionnaire (SRQ)

Annexe 4: Quality Assessment of Included Studies

4a: Quality Assessment of Cohort and Cross-sectional Studies

Study ID	Criteria														Overall Quality
	**1**	**2**	**3**	**4**	**5**	**6**	**7**	**8**	**9**	**10**	**11**	**12**	**13**	**14**	
1. Afridi 2014	Y	Y	Y	Y	Y	NA	N	Y	Y	N	Y	NA	Y	N	Low
2. Ahmad 2005	Y	Y	Y	Y	N	NA	N	Y	Y	N	Y	NA	Y	Y	Low
3. Ali 2009	Y	Y	Y	Y	N	Y	Y	Y	Y	N	Y	NA	N	Y	Low
4. Ali 2012	Y	Y	Y	Y	N	NA	NR	Y	Y	N	Y	NA	Y	Y	Low
5. Asad 2010	Y	Y	Y	Y	N	NA	NR	Y	Y	N	Y	NA	Y	Y	Low
6. Atif 2022	Y	Y	Y	Y	N	NA	Y	Y	Y	N	Y	NA	N	Y	Low
7. Ayub 2018	Y	Y	Y	Y	N	NA	NR	Y	Y	N	Y	NA	Y	Y	Low
8. Brown 2021	Y	Y	Y	Y	N	NA	Y	Y	Y	N	Y	NA	Y	Y	Moderate
9. Chung 2022	Y	Y	Y	Y	Y	Y	Y	Y	Y	N	Y	NA	Y	Y	Moderate
10. Din 2016	Y	Y	Y	Y	N	NA	NR	Y	Y	N	Y	NA	Y	Y	Low
11. Ghaffar 2017	Y	Y	Y	Y	Y	NA	NR	Y	Y	N	Y	NA	Y	Y	Moderate
12. Ghafoor 2021	Y	Y	Y	Y	Y	NA	NR	Y	cannot assess	N	PY	NA	Y	N	Low
13. Gul 2013	Y	Y	Y	Y	N	Y	N	Y	Y	N	Y	NA	N	N	Low
14. Gul 2017	Y	Y	Y	Y	Y	NA	NR	Y	Y	N	Y	NA	Y	N	Low
15. Habib 2019	Y	Y	Y	Y	Y	NA	Y	Y	Y	N	Y	NA	Y	N	Moderate
16. Habiba 2020	Y	Y	Y	Y	Y	NA	Y	Y	Y	N	Y	NA	Y	N	Moderate
17. Hamid 2008	Y	Y	Y	Y	N	NA	NR	Y	Y	N	Y	NA	Y	Y	Low
18. Hamirani 2006	Y	Y	Y	Y	N	NA	N	Y	Y	N	Y	NA	Y	N	Low
19. Humayun 2013	Y	Y	Y	Y	Y	NA	N	Y	Y	N	Y	NA	Y	N	Low
20. Husain 2006	Y	Y	Y	Y	N	NA	N	Y	Y	N	Y	NA	Y	N	Low
21. Husain 2011	Y	Y	Y	Y	N	NA	N	Y	Y	N	Y	NA	Y	Y	Low
22. Imran 2010	Y	Y	Y	Y	N	NA	N	Y	Y	N	Y	NA	Y	N	Low
23. Irum 2022	Y	Y	Y	Y	Y	NA	NR	Y	Y	N	Y	NA	Y	Y	Moderate
24. Ishtiaque 2020	Y	Y	Y	Y	N	NA	NR	Y	Y	N	Y	NA	Y	N	Low
25. Jabbar 2022	Y	Y	Y	Y	Y	NA	NR	Y	Y	N	Y	NA	Y	N	Low
26. Jamal 2018	Y	Y	Y	Y	Y	NA	NR	Y	Y	N	Y	NA	Y	N	Low
27. Kalar 2012	Y	Y	Y	Y	Y	NA	Y	Y	Y	N	Y	NA	Y	Y	High
28. Kalyani 2001	Y	Y	Y	Y	N	NA	N	Y	Y	N	Y	NA	Y	N	Low
29. Karmaliani 2009	Y	Y	Y	Y	Y	NA	N	Y	Y	N	Y	NA	Y	PY	Moderate
30. Kazi 2006	Y	Y	Y	Y	N	NA	N	Y	Y	N	Y	NA	Y	Y	Low
31. Khan 2020	Y	Y	Y	Y	N	NA	NR	Y	Y	N	Y	NA	Y	N	Low
32. Khan 2021	Y	Y	Y	Y	Y	NA	Y	Y	Y	N	Y	NA	Y	N	Moderate
33. Khanam 2011	Y	Y	Y	Y	N	NA	N	Y	Y	N	Y	NA	Y	N	Low
34. Khanam 2022	Y	Y	Y	Y	Y	Y	N	Y	Y	N	Y	NA	Y	Y	Moderate
35. LeMaster 2020	Y	Y	Y	Y	Y	Y	Y	Y	Y	N	Y	NA	Y	Y	Moderate
36. Maqbool 2022	Y	Y	Y	Y	Y	NA	NR	Y	Y	N	Y	NA	Y	Y	Moderate
37. a. Maselko 2018	Y	Y	Y	Y	Y	Y	cannot assess	Y	Y	N	Y	NA	Y	Y	High
38 b. Maselko 2019	Y	Y	Y	Y	N	Y	Y	Y	Y	N	Y	NA	Y	Y	Moderate

38. Masood 2017	Y	Y	Y	Y	N	NA	NR	Y	Y	N	Y	NA	Y	N	Low
39. Mir 2012	Y	Y	Y	Y	Y	NA	N	Y	Y	N	Y	NA	Y	Y	Moderate
40. Muneer 2009	Y	Y	Y	Y	N	NA	N	Y	Y	N	Y	NA	Y	N	Low
41. Niaz 2004	Y	Y	Y	Y	N	NA	NR	Y	Y	N	Y	NA	Y	N	Low
42. Noorullah 2020	Y	Y	Y	Y	Y	NA	Y	Y	Y	N	Y	NA	Y	N	Moderate
43. Premji 2020	Y	Y	Y	Y	PN	Y	N	Y	Y	N	Y	NA	Y	Y	Low
44. Rabia 2017	Y	Y	Y	Y	Y	NA	NR	Y	Y	N	Y	NA	Y	Y	Moderate
45. Rahman 2003	Y	Y	Y	Y	N	NA	N	Y	Y	N	Y	NA	Y	N	Low
46. Rahman 2007	Y	Y	Y	Y	N	NA	Y	Y	Y	N	Y	NA	Y	Y	Moderate
47. Ramji 2016	Y	Y	Y	Y	Y	NA	Y	Y	Y	N	Y	NA	Y	N	Moderate
48. Riaz 2020	Y	Y	Y	Y	PY	NA	N	Y	Y	N	Y	NA	Y	N	Low
49. Sabir 2019	Y	Y	Y	Y	PY	NA	NR	Y	Y	N	Y	NA	Y	N	Low
50. Sadaf 2011	Y	Y	Y	Y	N	NA	N	Y	Y	N	Y	NA	Y	N	Low
51. Sadiq 2016	Y	Y	Y	Y	N	NA	N	Y	Y	N	Y	NA	Y	N	Low
52. Saeed 2016	Y	Y	Y	Y	Y	NA	NR	Y	Y	N	Y	NA	Y	N	Low
53. Shah 2011	Y	Y	Y	Y	N	NA	Y	Y	Y	N	Y	NA	Y	Y	Moderate
54. Shah 2017	Y	Y	Y	Y	Y	NA	Y	Y	Y	N	Y	NA	Y	Y	Moderate
55. Shahid 2022	Y	Y	Y	Y	N	NA	NR	Y	Y	N	Y	NA	Y	N	Low
56. Shaikh 2011	Y	Y	Y	Y	Y	NA	NR	Y	Y	N	Y	NA	Y	N	Low
57. Tariq 2021	Y	Y	Y	Y	Y	Y	N	Y	Y	N	Y	NA	Y	N	Moderate
58. Waqas 2015	Y	Y	Y	Y	N	NA	NR	Y	Y	N	Y	NA	Y	Y	Low
59. Zahidie 2011	Y	Y	Y	Y	N	NA	N	Y	Y	N	Y	NA	Y	Y	Low
60. Zareen 2009	Y	Y	Y	Y	N	NA	NR	Y	Y	N	Y	NA	Y	Y	Low

Key:
Criteria
1. Was the research question or objective in this paper clearly stated?
2. Was the study population clearly specified and defined?
3. Was the participation rate of eligible persons at least 50%?
4. Were all the subjects selected or recruited from the same or similar populations (including the same time period)? Were inclusion and exclusion criteria for being in the study prespecified and applied uniformly to all participants?
5. Was a sample size justification, power description, or variance and effect estimates provided?
6. For the analyses in this paper, were the exposure(s) of interest measured prior to the outcome(s) being measured?
7. Was the timeframe sufficient so that one could reasonably expect to see an association between exposure and outcome if it existed?
8. For exposures that can vary in amount or level, did the study examine different levels of the exposure as related to the outcome (e.g., categories of exposure, or exposure measured as continuous variable)?
9. Were the exposure measures (independent variables) clearly defined, valid, reliable, and implemented consistently across all study participants?
10. Was the exposure(s) assessed more than once over time?
11. Were the outcome measures (dependent variables) clearly defined, valid, reliable, and implemented consistently across all study participants?
12. Were the outcome assessors blinded to the exposure status of participants?
13. Was loss to follow-up after baseline 20% or less?
14. Were key potential confounding variables measured and adjusted statistically for their impact on the relationship between exposure(s) and outcome(s)?

4b: Quality Assessment of Case-control Studies

	Criteria												Overall
**Study ID**	**1**	**2**	**3**	**4**	**5**	**6**	**7**	**8**	**9**	**10**	**11**	**12**	**Quality**
**1.** Khalid 1989	Y	Y	N	Y	cannot assess	Y	Y	N	Y	Y	NR	N	Low

Key
Criteria
1. Was the research question or objective in this paper clearly stated and appropriate?
2. Was the study population clearly specified and defined?
3. Did the authors include a sample size justification?
4. Were controls selected or recruited from the same or similar population that gave rise to the cases (including the same timeframe)?
5. Were the definitions, inclusion and exclusion criteria, algorithms or processes used to identify or select cases and controls valid, reliable, and implemented consistently across all study participants?
6. Were the cases clearly defined and differentiated from controls?
7. If less than 100 percent of eligible cases and/or controls were selected for the study, were the cases and/or controls randomly selected from those eligible?
8. Was there use of concurrent controls?
9. Were the investigators able to confirm that the exposure/risk occurred prior to the development of the condition or event that defined a participant as a case?
10. Were the measures of exposure/risk clearly defined, valid, reliable, and implemented consistently (including the same time period) across all study participants?
11. Were the assessors of exposure/risk blinded to the case or control status of participants?
12. Were key potential confounding variables measured and adjusted statistically in the analyses? If matching was used, did the investigators account for matching during study analysis?

Annexe 5: Risk Factors Associated with Maternal Perinatal Depression

**Maternal**			
**Outcomes**	**Antenatal Depression**	**Postnatal Depression**	**Perinatal Depression (Combined)**
**Age**			
<35 years	OR: 1.06; 95% CI: 0.47, 2.40; n=5; Heterogeneity: Tau^2 = 0.62; Chi^2 = 15.80, df = 4 (P = 0.003); I^2 = 75%	OR: 1.30; 95% CI: 0.89, 1.89; n=4; Heterogeneity: Tau^2 = 0.04; Chi^2 = 4.08, df = 3 (P = 0.25); I^2 = 27%	OR: 1.17; 95% CI: 0.76, 1.78; n=9; Heterogeneity: Tau^2 = 0.23; Chi^2 = 21.16, df = 8 (P = 0.007); I^2 = 62%
>35 years	OR 0.70; 95% CI:0.30, 1.66; n=4; Heterogeneity: Tau^2 = 0.41; Chi^2 = 7.09, df = 3 (P = 0.07); I^2 = 58%	OR: 0.89; 95% CI: 0.52, 1.55; n=5; Heterogeneity: Tau^2 = 0.27; Chi^2 = 17.45, df = 4 (P = 0.002); I^2 = 77%	OR: 0.82; 95% CI: 0.53, 1.27; n=9; Heterogeneity: Tau^2 = 0.25; Chi^2 = 25.16, df = 8 (P = 0.001); I^2 = 68%
**Age in years**	MD 0.90; 95% CI: -0.10, 1.89; n=3; Heterogeneity: Tau^2 = 0.42; Chi^2 = 4.34, df = 2 (P = 0.11); I^2 = 54%	MD -0.88; 95% CI: -2.28, 0.52; n=1	MD 0.60; 95% CI: -0.23, 1.43; n=5; Heterogeneity: Tau^2 = 0.47; Chi^2 = 9.28, df = 4 (P = 0.05); I^2 = 57%
**Residence**			
Rural	OR 1.69; 95% CI:0.69, 4.11; n= 1	**OR: 2.06; 95% CI: 1.03, 4.13; n=1**	**OR: 1.91; 95% CI: 1.11, 3.30; n=2; Heterogeneity: Tau^2 = 0.00; Chi^2 = 0.12, df = 1 (P = 0.73); I^2 = 0%**
Urban	OR 0.59; 95% CI:0.24, 1.44; n=1	**OR: 0.48; 95% CI: 0.24, 0.97; n=1**	**OR: 0.52; 95% CI: 0.30, 0.90; n=2; Heterogeneity: Tau^2 = 0.00; Chi^2 = 0.12, df = 1 (P = 0.73); I^2 = 0%**
**Education**			
Illiterate	**OR 2.01; 95% CI:1.56, 2.58; n= 5; Heterogeneity: Tau^2 = 0.00; Chi^2 = 1.89, df = 4 (P = 0.76); I^2 = 0%**	OR: 1.39; 95% CI: 0.73, 2.62; n=6; Heterogeneity: Tau^2 = 0.34; Chi^2 = 15.07, df = 4 (P = 0.005); I^2 = 73%	**OR: 1.56; 95% CI: 1.04, 2.34; n= 11; Heterogeneity: Tau^2 = 0.31; Chi^2 = 46.71, df = 10 (P < 0.00001); I^2 = 79%**
Literate	**OR 0.48; 95% CI:0.38, 0.62; n= 5; Heterogeneity: Tau^2 = 0.00; Chi^2 = 0.90, df = 4 (P = 0.92); I^2 = 0%**	OR: 1.13; 95% CI: 0.61, 2.09; n=5; Heterogeneity: Tau^2 = 0.50; Chi^2 = 34.16, df = 5 (P < 0.00001); I^2 = 85%	OR: 0.72; 95% CI: 0.51, 1.03; n=10; Heterogeneity: Tau^2 = 0.17; Chi^2 = 24.65, df = 9 (P = 0.003); I^2 = 63%
**Education in years**	MD -0.54; 95% CI: -1.99, 0.91; n=1		
**Education of family head/ husband**			
Illiterate	OR 0.97; 95% CI:0.13, 6.98; n= 2; Heterogeneity: Tau^2 = 1.34; Chi^2 = 2.09, df = 1 (P = 0.15); I^2 = 52%	**OR: 2.02; 95% CI: 1.24, 3.29; n=1**	**OR: 1.83; 95% CI: 1.25, 2.67; n=3; Heterogeneity: Tau^2 = 0.02; Chi^2 = 2.36, df = 2 (P = 0.31); I^2 = 15%**
Literate	**OR 0.55; 95% CI:0.36, 0.85; n=2; Heterogeneity: Tau^2 = 0.00; Chi^2 = 0.11, df = 1 (P = 0.74); I^2 = 0%**		
**Ethnicity**			
Urdu	OR 0.64; 95% CI:0.30, 1.35; n=1		
Other	OR 1.56; 95% CI:0.74, 3.29; n=1		
**Occupation**			
Housewife	OR 0.95; 95% CI:0.65, 1.40; n=3; Heterogeneity: Tau^2 = 0.00; Chi^2 = 1.51, df = 2 (P = 0.47); I^2 = 0%	OR: 0.60; 95% CI: 0.09, 3.96; n=4;	OR: 0.73; 95% CI: 0.24, 2.17; n= 7; Heterogeneity: Tau^2 = 2.01; Chi^2 = 138.14, df = 6 (P < 0.00001); I^2 = 96%
Working women	OR 1.05; 95% CI:0.72, 1.55; n=3; Heterogeneity: Tau^2 = 0.00; Chi^2 = 1.51, df = 2 (P = 0.47); I^2 = 0%	OR: 0.74; 95% CI: 0.13, 4.13; n=4	OR: 0.93; 95% CI: 0.40, 2.19; n=8; Heterogeneity: Tau^2 = 1.37; Chi^2 = 121.27, df = 7 (P < 0.00001); I^2 = 94%
**Husband's Occupation**			
Unemployed		**OR: 2.17; 95% CI: 1.19, 3.97; n=1**	**OR: 2.34; 95% CI: 1.51, 3.63; n=2; Heterogeneity: Tau^2 = 0.00; Chi^2 = 0.12, df = 1 (P = 0.72); I^2 = 0%**

Employed			**OR: 0.39; 95% CI: 0.21, 0.75; n=1**
**Husband is the only Breadwinner**			
Yes		**OR: 2.52; 95% CI: 1.28, 4.96; n=1**	
No		**OR: 0.40; 95% CI: 0.20, 0.78; n=1**	
**Type of family**			
Joint	OR 0.45; 95% CI:0.09, 2.38; n=3; Heterogeneity: Tau^2 = 2.06; Chi^2 = 43.02, df = 2 (P < 0.00001); I^2 = 95%	OR: 0.98; 95% CI: 0.56, 1.71; n=5; Heterogeneity: Tau^2 = 0.30; Chi^2 = 16.71, df = 4 (P = 0.002); I^2 = 76%	OR: 0.76; 95% CI: 0.41, 1.40; n=8; Heterogeneity: Tau^2 = 0.69; Chi^2 = 60.20, df = 7 (P < 0.00001); I^2 = 88%
Nuclear	OR 0.91; 95% CI:0.45, 1.82; n=; Heterogeneity: Tau^2 = 0.26; Chi^2 = 6.65, df = 2 (P = 0.04); I^2 = 70%	OR: 0.88; 95% CI: 0.43, 1.81; n=4; Heterogeneity: Tau^2 = 0.28; Chi^2 = 6.70, df = 2 (P = 0.04); I^2 = 70%	OR: 0.98; 95% CI: 0.68, 1.42; n= 7; Heterogeneity: Tau^2 = 0.15; Chi^2 = 18.61, df = 6 (P = 0.005); I^2 = 68%
**Number of people living in the house**			
Up to 5	OR 0.77; 95% CI:0.42, 1.41; n=1		
>5	OR 1.30; 95% CI:0.71, 2.40; n=1		
**Socioeconomic status**			
Upper	**OR 0.33; 95% CI: 0.18, 0.60; n=1**		OR: 0.60; 95% CI: 0.18, 1.98; n=2; Heterogeneity: Tau^2 = 0.63; Chi^2 = 6.63, df = 1 (P = 0.01); I^2 = 85%
Middle	OR 1.52; 95% CI: 0.91, 2.53; n=1		OR: 2.42; 95% CI: 0.95, 6.16; n= 2; Heterogeneity: Tau^2 = 0.38; Chi^2 = 5.77, df = 1 (P = 0.02); I^2 = 83%
Lower	OR 2.17; 95% CI: 0.66, 7.16; n=1		**OR: 2.67; 95% CI: 1.52, 4.67; n= 2; Heterogeneity: Tau^2 = 0.00; Chi^2 = 0.15, df = 1 (P = 0.70); I^2 = 0%**
**Monthly income**	MD $ -37.51; 95% CI: -94.32, 19.30; n=1	MD -773.17 PKR; 95% CI: -2014.33, 467.99; n=1	SMD -0.22; 95% CI: -0.49, 0.04; n=2; Heterogeneity: Tau^2 = 0.00; Chi^2 = 0.12, df = 1 (P = 0.73); I^2 = 0%
**Income**			
<10,000 PKR		OR: 1.21; 95% CI: 0.71, 2.05; n=2; Heterogeneity: Chi^2 = 2.03, df = 1 (P = 0.15); I^2 = 51%	OR: 1.01; 95% CI: 0.73, 1.39; n=3; Heterogeneity: Tau^2 = 0.00; Chi^2 = 2.03, df = 2 (P = 0.36); I^2 = 2%
>10,000 PKR		OR: 0.13; 95% CI: 0.01, 2.25; n=1	OR: 0.60; 95% CI: 0.10, 3.63; n= 2; Heterogeneity: Tau^2 = 1.11; Chi^2 = 2.06, df = 1 (P = 0.15); I^2 = 51%
<25,000 PKR	OR 1.25; 95% CI:0.54, 2.89; n=2; Heterogeneity: Tau^2 = 0.24; Chi^2 = 2.54, df = 1 (P = 0.11); I^2 = 61%		
>25,000 PKR	OR 0.22; 95% CI:0.01, 5.52; n=2; Heterogeneity: Tau^2 = 5.33; Chi^2 = 45.93, df = 1 (P < 0.00001); I^2 = 98%		
**Financially Independent**		**OR 0.45; 95% CI: 0.31, 0.67; n=1**	
**Family debt/ hardships**			
Yes	**OR 2.15; 95% CI:1.72, 2.70; n=2; Heterogeneity: Tau^2 = 0.00; Chi^2 = 0.29, df = 1 (P = 0.59); I^2 = 0%**	OR: 2.11; 95% CI: 0.84, 5.29; n=2; Heterogeneity: Tau^2 = 0.31; Chi^2 = 3.35, df = 1 (P = 0.07); I^2 = 70%	**OR: 2.20; 95% CI: 1.66, 2.90; n=4; Heterogeneity: Tau^2 = 0.02; Chi^2 = 3.67, df = 3 (P = 0.30); I^2 = 18%**
No	**OR 0.51; 95% CI:0.40, 0.64; n=1**	**OR: 0.30; 95% CI: 0.16, 0.58; n=1**	**OR: 0.43; 95% CI: 0.27, 0.68; n=2; Heterogeneity: Tau^2 = 0.07; Chi^2 = 2.15, df = 1 (P = 0.14); I^2 = 53%**

**Poverty (<3 on 5-point scale)**		OR: 1.47; 95% CI: 0.97, 2.22; n=1	
**Food insecurity**			
Yes	**OR 3.45; 95% CI:2.59, 4.60; n=2; Heterogeneity: Tau^2 = 0.00; Chi^2 = 0.85, df = 1 (P = 0.36); I^2 = 0%**		
No	**OR 0.30; 95% CI:0.23, 0.40; n=2; Heterogeneity: Tau^2 = 0.00; Chi^2 = 0.36, df = 1 (P = 0.55); I^2 = 0%**		
**Household decision maker**			
Self/ husband	OR 1.32; 95% CI:0.70, 2.50; n=1		
In-laws	OR 1.47; 95% CI:0.74, 2.95; n=1		
Combined	OR 0.55; 95% CI:0.29, 1.04; n=1		
**Willingness of pregnancy**			
Yes	OR 1.14; 95% CI:0.39, 3.31; n=1		
No	OR 0.88; 95% CI:0.30, 2.53; n=1		
**Pregnancy-related factors**<br><br>**Nature of pregnancy**			
Unplanned	OR 1.24; 95% CI:0.60, 2.57; n=4; Heterogeneity: Tau^2 = 0.73; Chi^2 = 32.84, df = 3 (P < 0.00001); I^2 = 91%	OR: 3.09; 95% CI: 0.83, 11.41; n=1	OR: 1.43; 95% CI: 0.82, 2.49; n= 6; Heterogeneity: Tau^2 = 0.37; Chi^2 = 29.06, df = 5 (P < 0.0001); I^2 = 83%
Planned	**OR 0.35; 95% CI:0.14, 0.85; n=4; Heterogeneity: Tau^2 = 0.49; Chi^2 = 26.96, df = 3 (P < 0.00001); I^2 = 89%**		OR: 0.57; 95% CI: 0.19, 1.70; n= 5; Heterogeneity: Tau^2 = 1.42; Chi^2 = 78.03, df = 4 (P < 0.00001); I^2 = 95%
**Gestational weeks**	MD -0.20; 95% CI: -0.85, 0.45; n=1		MD -0.15; 95% CI: -0.70, 0.39; n=2; Heterogeneity: Tau^2 = 0.00; Chi^2 = 0.07, df = 1 (P = 0.79); I^2 = 0%
**Type of delivery**			
Vaginal		OR: 1.24; 95% CI: 0.60, 2.59; n=3; Heterogeneity: Tau^2 = 0.22; Chi^2 = 4.24, df = 2 (P = 0.12); I^2 = 53%	
C-section		OR: 0.83; 95% CI: 0.48, 1.45; n=3; Heterogeneity: Tau^2 = 0.13; Chi^2 = 4.55, df = 2 (P = 0.10); I^2 = 56%	
**Number of previous vaginal deliveries**			
Never	OR 2.15; 95% CI:0.88, 5.27; n=1		
Yes	OR 0.46; 95% CI:0.19, 1.14; n=1		
**Number of previous C-sections**			
Never	OR 0.41; 95% CI:0.01, 15.90; n=2; Heterogeneity: Tau^2 = 6.69; Chi^2 = 23.42, df = 1 (P < 0.00001); I^2 = 96%		
Yes	**OR 0.50; 95% CI:0.28, 0.91; n=2; Heterogeneity: Tau^2 = 0.00; Chi^2 = 0.28, df = 1 (P = 0.59); I^2 = 0%**		

**Parity**			
Primigravida	OR 0.78; 95% CI:0.30, 2.04; n=5; Heterogeneity: Tau² = 0.97; Chi² = 24.12, df = 4 (P < 0.0001); I² = 83%	OR: 0.82; 95% CI: 0.29, 2.33; n=2; Heterogeneity: Tau² = 0.43; Chi² = 4.07, df = 1 (P = 0.04); I² = 75%	OR: 0.96; 95% CI: 0.51, 1.79; n=8; Heterogeneity: Tau² = 0.63; Chi² = 35.56, df = 7 (P < 0.00001); I² = 80%
Multigravida	OR 0.48; 95% CI:0.04, 6.17; n=2; Heterogeneity: Tau² = 3.30; Chi² = 32.24, df = 1 (P < 0.00001); I² = 97%	OR: 1.13; 95% CI: 0.49, 2.62; n=2; Heterogeneity: Tau² = 0.25; Chi² = 3.10, df = 1 (P = 0.08); I² = 68%	OR: 1.04; 95% CI: 0.35, 3.09; n= 5; Heterogeneity: Tau² = 1.43; Chi² = 58.03, df = 4 (P < 0.00001); I² = 93%
Nulliparous	**OR 0.43; 95% CI:0.26, 0.72; n=4; Heterogeneity: Tau² = 0.17; Chi² = 9.45, df = 3 (P = 0.02); I² = 68%**	**OR: 5.30; 95% CI: 1.31, 21.47; n=1**	**OR: 0.60; 95% CI: 0.38, 0.95; n= 6; Heterogeneity: Tau² = 0.21; Chi² = 25.33, df = 5 (P = 0.0001); I² = 80%**
Primiparous		OR: 0.25; 95% CI: 0.03, 2.33; n=3; Heterogeneity: Tau² = 3.65; Chi² = 43.05, df = 2 (P < 0.00001); I² = 95%	
Multiparous	**OR 2.14; 95% CI:1.14, 4.01; n=5; Heterogeneity: Tau² = 0.33; Chi² = 14.52, df = 4 (P = 0.006); I² = 72%**	OR: 0.53; 95% CI: 0.11, 2.52; n=4; Heterogeneity: Tau² = 2.46; Chi² = 96.53, df = 3 (P < 0.00001); I² = 97%	OR: 1.16; 95% CI: 0.64, 2.09; n=10; Heterogeneity: Tau² = 0.77; Chi² = 126.77, df = 9 (P < 0.00001); I² = 93%
**Pregnancy duration**			
1st trimester	OR 0.36; 95% CI:0.02, 5.27; n=2; Heterogeneity: Tau² = 3.58; Chi² = 20.07, df = 1 (P < 0.00001); I² = 95%		
2nd trimester	**OR 0.52; 95% CI:0.31, 0.86; n=4; Heterogeneity: Tau² = 0.16; Chi² = 7.25, df = 3 (P = 0.06); I² = 59%**		
3rd trimester	OR 1.21; 95% CI:0.60, 2.47; n=4; Heterogeneity: Tau² = 0.41; Chi² = 14.14, df = 3 (P = 0.003); I² = 79%		
**Antenatal Care Received**			
Yes		OR: 0.32; 95% CI: 0.11, 0.92; n=1	
No		**OR: 3.09; 95% CI: 1.09, 8.82; n=1**	
**Intentions of using family planning**			
Yes	OR 0.85; 95% CI:0.46, 1.55; n=1		
No	OR 1.07; 95% CI:0.57, 2.04; n=1		
Not planned yet	OR 1.25; 95% CI:0.51, 3.08; n=1		
**Could Decide About Family Planning Method Used**			
Yes	OR 0.95; 95% CI:0.48, 1.91; n=1		
No	OR 1.02; 95% CI:0.56, 1.88; n=1		
Not planned yet	OR 1.02; 95% CI:0.52, 2.00; n=1		
**Contraception/ ever used contraception**			
Yes	**OR 1.56; 95% CI:1.07, 2.29; n=2; Heterogeneity: Tau² = 0.00; Chi² = 0.79, df = 1 (P = 0.37); I² = 0%**		**OR: 1.52; 95% CI: 1.08, 2.15; n=3; Heterogeneity: Tau² = 0.00; Chi² = 0.89, df = 2 (P = 0.64); I² = 0%**
No	OR 0.57; 95% CI:0.30, 1.07; n=2; Heterogeneity: Tau² = 0.13; Chi² = 2.43, df = 1 (P = 0.12); I² = 59%		OR: 0.91; 95% CI: 0.33, 2.50; n=3; Heterogeneity: Tau² = 0.71; Chi² = 18.83, df = 2 (P < 0.0001); I² = 89%
**Fear of childbirth**			

Yes	OR 0.94; 95% CI:0.35, 2.57; n=2; Heterogeneity: Tau² = 0.45; Chi² = 6.74, df = 1 (P = 0.009); I² = 85%		
No	OR 0.21; 95% CI:0.02, 2.02; n=2; Heterogeneity: Tau² = 2.55; Chi² = 28.91, df = 1 (P < 0.00001); I² = 97%		
**Family/Spouse Support**<br><br>**Spouse support**			
Yes	OR 0.66; 95% CI:0.23, 1.89; n=2; Heterogeneity: Tau² = 0.43; Chi² = 3.71, df = 1 (P = 0.05); I² = 73%	OR: 0.05; 95% CI: 0.01, 0.41; n=1	OR: 0.36; 95% CI: 0.09, 1.44; n=3; Heterogeneity: Tau² = 1.12; Chi² = 10.71, df = 2 (P = 0.005); I² = 81%
No	OR 1.49; 95% CI:0.50, 4.40; n=2; Heterogeneity: Tau² = 0.47; Chi² = 3.96, df = 1 (P = 0.05); I² = 75%	OR: 18.75; 95% CI: 2.45, 143.63; n=1	OR: 2.73; 95% CI: 0.67, 11.07; n=3; Heterogeneity: Tau² = 1.17; Chi² = 11.09, df = 2 (P = 0.004); I² = 82%
**Husband away from home**			
Yes		OR: 0.86; 95% CI: 0.54, 1.38; n=1	OR: 1.12; 95% CI: 0.69, 1.83; n=2; Heterogeneity: Tau² = 0.07; Chi² = 2.52, df = 1 (P = 0.11); I² = 60%
**Presence of husband – Can skip**			
Always	OR 1.23; 95% CI:0.74, 2.05; n=1		
Weekends	OR 0.77; 95% CI:0.29, 2.04; n=1		
**Separation from husband**			
Yes	OR 7.53; 95% CI:0.38, 147.73; n=2; Heterogeneity: Tau² = 3.66; Chi² = 4.17, df = 1 (P = 0.04); I² = 76%		
No	**OR 0.18; 95% CI:0.04, 0.74; n=2; Heterogeneity: Tau² = 0.90; Chi² = 6.40, df = 1 (P = 0.01); I² = 84%**		
**Marital problems**	**OR 7.57; 95% CI:2.62, 21.85; n=1**	OR: 4.34; 95% CI: 0.34, 56.07; n=2; Heterogeneity: Tau² = 3.12; Chi² = 11.65, df = 1 (P = 0.0006); I² = 91%	**OR: 5.31; 95% CI: 1.21, 23.31; n=3; Heterogeneity: Tau² = 1.42; Chi² = 11.86, df = 2 (P = 0.003); I² = 83%**
**Quality of marital relationship**			
Fair	**OR 0.19; 95% CI:0.08, 0.43; n=1**		
Poor	**OR 5.40; 95% CI:2.30, 12.65; n=1**		
**Major Arguments/Relationship Difficulties or Problems**			
Yes	OR 3.07; 95% CI:0.95, 9.90; n=2; Heterogeneity: Tau² = 0.60; Chi² = 6.40, df = 1 (P = 0.01); I² = 84%	OR: 0.52; 95% CI: 0.14, 1.96; n=1	OR: 1.89; 95% CI: 0.56, 6.32; n=3; Heterogeneity: Tau² = 0.94; Chi² = 13.12, df = 2 (P = 0.001); I² = 85%
No	OR 0.60; 95% CI:0.30, 1.20; n=1		OR: 0.60; 95% CI: 0.30, 1.20; n=1
**Domestic Violence/IPV**			

Yes	OR 1.90; 95% CI:0.56, 6.47; n=5; Heterogeneity: $Tau^2 = 1.67$; $Chi^2 = 45.86$, df = 4 ($P < 0.00001$); $I^2 = 91\%$	OR: 13.38; 95% CI: 0.68, 264.05; n=1	OR: 2.26; 95% CI: 0.70, 7.30; n=6; Heterogeneity: $Tau^2 = 1.68$; $Chi^2 = 47.90$, df = 5 ($P < 0.00001$); $I^2 = 90\%$
No	**OR 0.25; 95% CI:0.07, 0.95; n=5; Heterogeneity: $Tau^2 = 1.99$; $Chi^2 = 48.64$, df = 4 ($P < 0.00001$); $I^2 = 92\%$**		
**Unhappy/Problems with parents/in-laws**			
Yes	**OR 4.08; 95% CI:2.36, 7.04; n=2; Heterogeneity: $Tau^2 = 0.00$; $Chi^2 = 0.00$, df = 1 ($P = 0.98$); $I^2 = 0\%$**	OR: 1.62; 95% CI: 1.01, 2.61; n=2; Heterogeneity: $Tau^2 = 0.00$; $Chi^2 = 0.79$, df = 1 ($P = 0.37$); $I^2 = 0\%$	**OR: 2.37; 95% CI: 1.32, 4.27; n=4; Heterogeneity: $Tau^2 = 0.20$; $Chi^2 = 7.05$, df = 3 ($P = 0.07$); $I^2 = 57\%$**
No	**OR 0.24; 95% CI:0.14, 0.44; n=1**	**OR: 0.52; 95% CI: 0.28, 0.95; n=1**	**OR: 0.35; 95% CI: 0.17, 0.74; n=2; Heterogeneity: $Tau^2 = 0.19$; $Chi^2 = 3.00$, df = 1 ($P = 0.08$); $I^2 = 67\%$**
**Housing difficulties**	**OR 2.81; 95% CI:1.70, 4.63; n=1**	OR: 1.47; 95% CI: 0.51, 4.21; n=1	**OR: 2.41; 95% CI: 1.41, 4.13; n=2; Heterogeneity: $Tau^2 = 0.03$; $Chi^2 = 1.18$, df = 1 ($P = 0.28$); $I^2 = 16\%$**
**Feels Happy and Comfortable at Home**			
Yes		**OR: 0.08; 95% CI: 0.02, 0.37; n=1**	
No		**OR: 11.82; 95% CI: 2.69, 51.97; n=1**	
**Infant grandmother lives with family**		**OR: 0.49; 95% CI: 0.33, 0.74; n=1**	
**Daily physical help in childcare by at least 1 family member**			
Yes		**OR: 0.38; 95% CI: 0.27, 0.54; n=2; Heterogeneity: $Tau^2 = 0.00$; $Chi^2 = 0.53$, df = 1 ($P = 0.47$); $I^2 = 0\%$**	
No		**OR: 3.23; 95% CI: 1.70, 6.14; n=1**	
**Prohibited to do household work during puerperium**			
Yes		**OR: 0.32; 95% CI: 0.15, 0.68; n=1**	
No		**OR: 3.15; 95% CI: 1.46, 6.77; n=1**	
**Had difficulty meeting daily needs**		OR: 0.92; 95% CI: 0.65, 1.30; n=1	
**Able to complete 'chilla' period**		**OR: 0.48; 95% CI: 0.33, 0.70; n=1**	
**Problems with neighbours**		OR: 2.53; 95% CI: 0.54, 11.76; n=1	
**Work related problems**		OR: 1.83; 95% CI: 0.75, 4.50; n=1	
**Legal problems**		OR: 5.51; 95% CI: 0.22, 137.77; n=1	
**Stressful life events**			

**Death of Some Close Relative (within 1 year)**			
Yes	**OR 1.97; 95% CI:1.24, 3.14; n=1**		
No	**OR 0.51; 95% CI:0.32, 0.81; n=1**		
**Loss in in-laws**			
Yes	OR 0.81; 95% CI:0.51, 1.29; n=1		
No	OR 1.19; 95% CI:0.75, 1.88; n=1		
**Loss of parents/mother**			
Yes	**OR 1.81; 95% CI:1.11, 2.97; n=1**	OR: 2.26; 95% CI: 0.83, 6.15; n=1	**OR: 1.89; 95% CI: 1.22, 2.95; n=2; Heterogeneity: $Tau^2 = 0.00$; $Chi^2 = 0.15$, df = 1 (P = 0.70); $I^2 = 0\%$**
No	**OR 0.55; 95% CI:0.34, 0.90; n=1**		**OR: 0.55; 95% CI: 0.34, 0.90; n=1**
**Other stressful life events**			
Yes	OR 2.05; 95% CI:0.93, 4.51; n=1		
No	**OR 0.05; 95% CI:0.03, 0.11; n=1**		
**Emotional reaction**			
Unwelcome		**OR: 4.97; 95% CI: 2.93, 8.42; n=1**	
Welcome		**OR: 0.20; 95% CI: 0.12, 0.34; n=1**	
**Lack of friend or confident**	OR 0.85; 95% CI:0.56, 1.28; n=1		
**Satisfied with life**			
Yes	**OR 0.22; 95% CI:0.06, 0.81; n=1**		
No	**OR 4.56; 95% CI:1.24, 16.82; n=1**		
**Social role change**	OR 1.28; 95% CI:0.89, 1.85; n=1		
**Significant Other Made Redundant**	**OR 3.02; 95% CI:1.75, 5.21; n=1**		
**Passive smoker**			
Yes	**OR 2.68; 95% CI:1.73, 4.17; n=1**		
No	**OR 0.37; 95% CI:0.24, 0.58; n=1**		
**Coping mechanism**<br><br>**Drug abuse**			
Yes	**OR 2.48; 95% CI:1.09, 5.62; n=2; Heterogeneity: $Tau^2 = 0.00$; $Chi^2 = 0.59$, df = 1 (P = 0.44); $I^2 = 0\%$**		

No	**OR 0.40; 95% CI:0.18, 0.92; n=2; Heterogeneity: Tau² = 0.00; Chi² = 0.59, df = 1 (P = 0.44); I² = 0%**		
**Body mass index**			
Underweight	OR 0.19; 95% CI:0.01, 3.89; n=1		
Normal	OR 0.72; 95% CI:0.36, 1.45; n=2; Heterogeneity: Tau² = 0.16; Chi² = 2.77, df = 1 (P = 0.10); I² = 64%		
Overweight	OR 1.14; 95% CI:0.76, 1.70; n=2; Heterogeneity: Tau² = 0.00; Chi² = 0.11, df = 1 (P = 0.75); I² = 0%		
Obese	OR 2.84; 95% CI:0.14, 55.98; n=2; Heterogeneity: Tau² = 3.71; Chi² = 4.29, df = 1 (P = 0.04); I² = 77%		
**Body mass index**	MD 0.00; 95% CI: -1.87, 1.87; n=1		
**Weight (kgs)**	MD 0.50; 95% CI: -5.05, 6.05; n=1		
**Height (cm)**			
144-154	OR 1.22; 95% CI:0.42, 3.52; n=1		
155-164	OR 1.03; 95% CI:0.42, 2.48; n=1		
165-175	OR 0.82; 95% CI:0.32, 2.12; n=1		
**Regular physical activity**			
Yes	**OR 0.43; 95% CI:0.20, 0.89; n=1**		
No	**OR 2.34; 95% CI:1.12, 4.90; n=1**		
**Healthy eating index**			
Poor	**OR 6.54; 95% CI:2.47, 17.30; n=1**		
Moderate	**OR 0.15; 95% CI:0.06, 0.40; n=1**		
**Bereavement/Illness**	OR 1.34; 95% CI:0.88, 2.03; n=1	OR: 2.04; 95% CI: 0.98, 4.24; n=1	**OR: 1.49; 95% CI: 1.03, 2.13; n=2; Heterogeneity: Tau² = 0.00; Chi² = 0.95, df = 1 (P = 0.33); I² = 0%**
**Major illness in family**	OR 1.37; 95% CI:0.94, 2.00; n=1		
**Previous medical history**<br><br>**Family history**			
Depression/ psychiatric illness	OR 1.35; 95% CI:0.43, 4.31; n=3; Heterogeneity: Tau² = 0.93; Chi² = 19.89, df = 2 (P < 0.0001); I² = 90%		
Pre-eclampsia	**OR 2.10; 95% CI:1.37, 3.20; n=1**		
Diabetes	OR 1.41; 95% CI:0.95, 2.09; n=1		
Congenital anomaly	OR 0.97; 95% CI:0.59, 1.60; n=1		

**History of Abortion/Miscarriage /Intrauterine Death**			
Yes	OR 1.30; 95% CI:0.83, 2.06; n=3; Heterogeneity: $Tau^2 = 0.09$; $Chi^2 = 4.40$, df = 2 (P = 0.11); $I^2 = 55\%$		
No	**OR 0.19; 95% CI:0.04, 0.98; n=3; Heterogeneity: $Tau^2 = 1.94$; $Chi^2 = 46.07$, df = 2 (P < 0.00001); $I^2 = 96\%$**		
**Previous psychiatric illness/ psychiatric drug use**			
Yes	**OR 2.57; 95% CI:1.04, 6.35; n=2; Heterogeneity: $Tau^2 = 0.23$; $Chi^2 = 2.17$, df = 1 (P = 0.14); $I^2 = 54\%$**	**OR: 7.14; 95% CI: 3.54, 14.37; n=1**	**OR: 3.42; 95% CI: 1.56, 7.51; n=4; Heterogeneity: $Tau^2 = 0.36$; $Chi^2 = 7.49$, df = 3 (P = 0.06); $I^2 = 60\%$**
No	**OR 0.42; 95% CI:0.20, 0.87; n=2; Heterogeneity: $Tau^2 = 0.09$; $Chi^2 = 1.46$, df = 1 (P = 0.23); $I^2 = 31\%$**	**OR: 0.14; 95% CI: 0.07, 0.28; n=1**	**OR: 0.29; 95% CI: 0.12, 0.68; n=3; Heterogeneity: $Tau^2 = 0.42$; $Chi^2 = 6.98$, df = 2 (P = 0.03); $I^2 = 71\%$**
**Ever Self Treated or Any Family Member for Psychiatric Disorder**			
Yes	OR 1.99; 95% CI:0.82, 4.80; n=1		
No	OR 0.50; 95% CI:0.21, 1.21; n=1		
**Ever Seek Help for Reducing Worry**			
Yes	OR 0.59; 95% CI:0.24, 1.45; n=1		
No	OR 1.69; 95% CI:0.69, 4.15; n=1		
**Childhood traumatic event**			
Yes	OR 2.15; 95% CI:0.52, 8.89; n=1		
No	**OR 0.15; 95% CI:0.07, 0.29; n=1**		
**Adverse Life Events in a Year Before Pregnancy**			
Yes	OR 1.57; 95% CI:0.90, 2.74; n=1		
No	OR 0.64; 95% CI:0.36, 1.11; n=1		
**Obstetric complications**			
Yes		OR: 1.34; 95% CI: 0.61, 2.95; n=1	OR: 1.51; 95% CI: 0.95, 2.41; n=3; Heterogeneity: $Tau^2 = 0.00$; $Chi^2 = 0.93$, df = 4 (P = 0.92); $I^2 = 0\%$
No		OR: 0.75; 95% CI: 0.34, 1.65; n=1	OR: 0.73; 95% CI: 0.45, 1.19; n=3; Heterogeneity: $Tau^2 = 0.00$; $Chi^2 = 1.56$, df = 4 (P = 0.82); $I^2 = 0\%$
**Adverse pregnancy outcomes**			

Yes	OR 1.61; 95% CI:0.90, 2.88; n=2; Heterogeneity: Tau² = 0.00; Chi² = 0.79, df = 3 (P = 0.85); I² = 0%		
No	OR 0.73; 95% CI:0.40, 1.33; n=2; Heterogeneity: Tau² = 0.00; Chi² = 1.56, df = 3 (P = 0.67); I² = 0%		
**Pregnancy-related low backpain (PLBP)**			
Yes	OR 1.06; 95% CI:0.41, 2.73; n=1		
No	OR 0.94; 95% CI:0.37, 2.43; n=1		
**Pregnancy induced hypertension**			
Yes		**OR: 3.05; 95% CI: 1.38, 6.73; n=1**	
No		**OR: 0.33; 95% CI: 0.15, 0.73; n=1**	
**Medical complications**			
Yes		OR: 1.29; 95% CI: 0.62, 2.69; n=1	
No		OR: 0.78; 95% CI: 0.37, 1.62; n=1	
**Social/cultural -**<br><br>**Baby Sex**			
Boy		OR: 0.74; 95% CI: 0.46, 1.21; n=1	
Girl		OR: 1.35; 95% CI: 0.83, 2.19; n=1	
**Pressure From Family for a Male Child**			
Yes	OR 1.40; 95% CI:0.81, 2.44; n=1		
No	OR 0.71; 95% CI:0.41, 1.24; n=1		
**Had one or more female child**		OR: 1.45; 95% CI: 0.56, 3.71; n=2; Heterogeneity: Tau² = 0.42; Chi² = 11.30, df = 1 (P = 0.0008); I² = 91%	
**Infant had trouble breastfeeding**		OR: 0.93; 95% CI: 0.66, 1.32; n=1	
**Infant fed formula**		OR: 1.01; 95% CI: 0.71, 1.42; n=1	
**Infant had difficulty sucking**		OR: 1.14; 95% CI: 0.81, 1.61; n=1	
**Infant wanted to be carried all times**		OR: 1.19; 95% CI: 0.85, 1.68; n=1	
**Infant had fever**		OR: 0.74; 95% CI: 0.53, 1.05; n=1	
**How child's disease affected relationship with husband**			
Has become better		**OR: 0.22; 95% CI: 0.06, 0.81; n=1**	

Unaffected		OR: 0.62; 95% CI: 0.29, 1.36; n=1	
Deteriorated		**OR: 9.25; 95% CI: 2.08, 41.12; n=1**	
**How child's disease affected relations with social circle, friends, and relatives**			
Has become better		OR: 0.39; 95% CI: 0.04, 4.43; n=1	
Unaffected		**OR: 0.11; 95% CI: 0.04, 0.29; n=1**	
Deteriorated		**OR: 15.52; 95% CI: 4.56, 52.90; n=1**	
**Mother blamed for child disability**			
Yes		**OR: 3.48; 95% CI: 1.80, 6.71; n=1**	
No		**OR: 0.29; 95% CI: 0.15, 0.55; n=1**	
**COVID Related Factors:**			
**Perceived Vulnerability of COVID**			
Yes	**OR 3.03; 95% CI:1.39, 6.61; n=1**		
No	**OR 0.33; 95% CI:0.15, 0.72; n=1**		
**Concerns About Getting Prenatal care**			
Yes	OR 1.82; 95% CI:0.89, 3.73; n=1		
No	OR 0.55; 95% CI:0.27, 1.12; n=1		
**Financial Problems During Pandemic**			
Yes	OR 0.81; 95% CI:0.39, 1.66; n=1		
No	OR 1.24; 95% CI:0.60, 2.54; n=1		
**Remained in Isolation During This Pregnancy**			
Yes	**OR 0.31; 95% CI:0.15, 0.65; n=1**		
No	**OR 10.08; 95% CI:4.53, 22.45; n=1**		
**Fear That Baby Might Get Infected After Birth**			
Yes	**OR 2.05; 95% CI:1.00, 4.23; n=1**		
No	OR 0.49; 95% CI:0.24, 1.00; n=1		

Annexe 6: Risk Factors Associated with Paternal Postnatal Depression

Paternal Post-natal Depression	
**Outcomes**	**Estimate**
**Age**	
<30 years	**OR: 11.25; 95% CI: 2.13, 59.38; n=1**
>= 30 years	**OR: 0.15; 95% CI: 0.03, 0.65; n=1**
**Employment**	
Employed	**OR: 0.08; 95% CI: 0.01, 0.85; n=1**
Unemployed	**OR: 12.67; 95% CI: 1.18, 136.44; n=1**
**Financial hardships**	
Yes	**OR: 8.70; 95% CI: 1.96, 38.65; n=1**
No	**OR: 0.11; 95% CI: 0.03, 0.51; n=1**
**Number of children**	
Only 1 child (including newborn)	OR: 0.60; 95% CI: 0.14, 2.57; n=1
Multiple children (including newborn)	OR: 1.68; 95% CI: 0.39, 7.24; n=1
**Satisfaction with Marital Relationship**	
Satisfied	OR: 0.25; 95% CI: 0.06, 1.07; n=1
Unsatisfied	OR: 3.93; 95% CI: 0.93, 16.58; n=1
**Sleep Disturbance**	
Yes	**OR: 11.63; 95% CI: 2.54, 53.17; n=1**
No	**OR: 0.09; 95% CI: 0.02, 0.39; n=1**
**Spouse Sleep Disturbance**	
Yes	**OR: 5.36; 95% CI: 1.24, 23.10; n=1**
No	**OR: 0.19; 95% CI: 0.04, 0.80; n=1**
**Spouse Depression status**	
Yes	**OR: 11.25; 95% CI: 2.13, 59.38; n=1**
No	**OR: 0.09; 95% CI: 0.02, 0.47; n=1**

Annexe 7: Forest on the Prevalence of Paternal Postnatal Depression

Study	Events	Total	Weight, F-T, Random, 95% CI	
Atif 2022 (3 months postnatal; EPDS: >10)	12	21	44.34%	0.571 [0.353, 0.778]
Noorullah 2020 (6 weeks to 1 year post childbirth; EPDS >10)	34	120	55.66%	0.283 [0.206, 0.368]
**Total (95% CI)**	**46**	**141**		0.405 [0.149, 0.690]

Heterogeneity: τ^2=0.04, χ^2=6.16, df=1 (P= 0.013) I^2=83.8

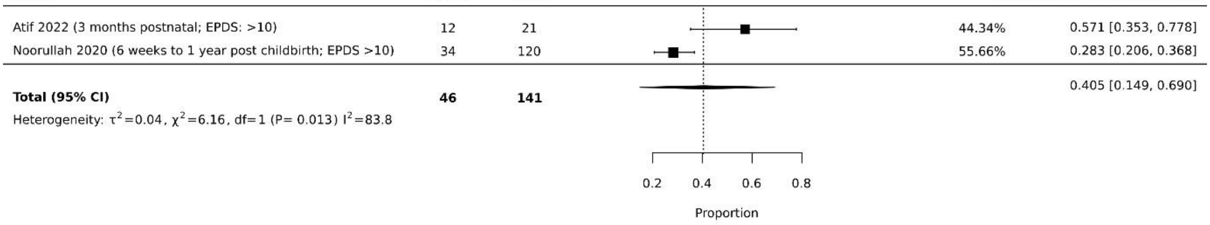

References

1. Afridi F, Batool I, Jabbar S, et al. Frequency of postnatal depression at a tertiary care hospital. *Journal of Medical Sciences* 2014;22(1):35-38.
2. Ahmad I, Khan M. Risk factors associated with post-natal depression in Pakistani women. *Pakistan Journal of Social and Clinical Psychology* 2005;3(1-2):41-50.
3. Ali NS, Ali BS, Azam IS. Post partum anxiety and depression in peri-urban communities of Karachi, Pakistan: a quasi-experimental study. *BMC public health* 2009;9:1-10.
4. Ali NS, Azam IS, Ali BS, et al. Frequency and associated factors for anxiety and depression in pregnant women: a hospital-based cross-sectional study. *The Scientific World Journal* 2012;2012
5. Asad N, Karmaliani R, Sullaiman N, et al. Prevalence of suicidal thoughts and attempts among pregnant Pakistani women. *Acta obstetricia et gynecologica Scandinavica* 2010;89(12):1545-51.
6. Atif M, Halaki M, Chow CM, et al. Risk factors of paternal postnatal depression in Pakistan: Findings from an urban sample. *Nursing & health sciences* 2022;24(3):618-24. doi: https://dx.doi.org/10.1111/nhs.12954
7. Ayyub H, Sarfraz M, Mir K, et al. Association of antenatal depression and household food insecurity among pregnant women: a cross-sectional study from slums of Lahore. *Journal of Ayub Medical College Abbottabad* 2018;30(3):366-71.
8. Brown RH, Eisner M, Walker S, et al. The impact of maternal adverse childhood experiences and prenatal depressive symptoms on foetal attachment: Preliminary evidence from expectant mothers across eight middle-income countries. *Journal of Affective Disorders* 2021;295:612-19.
9. Chung EO, Hagaman A, Bibi A, et al. Mother-in-law childcare and perinatal depression in rural Pakistan. *Women's Health* 2022;18 doi: https://dx.doi.org/10.1177/17455057221141288
10. Din Zu, Ambreen S, Iqbal Z, et al. Determinants of Antenatal Psychological Distress in Pakistani Women/Pakistanli Kadinlarda Antenatal Psikolojik Stresi Etkileyen Faktörler. *Noro-Psikyatri Arsivi* 2016;53(2):152.
11. Ghaffar R, Iqbal Q, Khalid A, et al. Frequency and predictors of anxiety and depression among pregnant women attending tertiary healthcare institutes of Quetta City, Pakistan. *BMC women's health* 2017;17:1-8.
12. Ghafoor S, Aftab S, Baloch ZH, et al. Postpartum depression & body image dissatisfaction in housewives and working women. *Pakistan Journal of Medical and Health Sciences* 2021;15(4):1219-23.
13. Gul M, Bajwa S, Niaz S, et al. Postnatal depression and its comparison with the gender of newborn in fourth pregnancy. *International Journal of Culture and Mental Health* 2013;6(1):16-28.
14. Gul F, Sherin A, Jabeen M, et al. Association of stress with anxiety and depression during pregnancy. *Journal of the Pakistan Medical Association* 2017;67(12):1803-08.
15. Habib H, Ali W. Psychosocial correlates of prolonged postpartum depression in mothers of children with movement disorders: cross-sectional study from a paediatric developmental rehabilitation centre in Peshawar. *JPMA The Journal of the Pakistan Medical Association* 2019;69(9):1293-99.
16. Habiba U, Rana MS, Hanif A, et al. Prevalence and risk factors associated with prenatal depression among pregnant women in Faisalabad, Pakistan. *Pakistan journal of pharmaceutical sciences* 2020;33(5):2355-60. doi: https://dx.doi.org/10.36721/PJPS.2020.33.5.SUP.2355-2360.1
17. Hamid F, Asif A, Haider II. Study of anxiety and depression during pregnancy. *Pak J Med Sci* 2008;24(6):861-4.

18. Hamirani MM, Sultana A, Ibrahim Z, et al. Frequency of prenatal depression in second and third trimesters of pregnancy in Karachi: a hospital based study. *Journal of the Liaquat University of Medical and Health Sciences* 2006;5(3):106-09.
19. Humayun A, Haider I, Imran N, et al. Antenatal depression and its predictors in Lahore, Pakistan. *EMHJ-Eastern Mediterranean Health Journal, 19 (4), 327-332, 2013* 2013
20. Husain N, Bevc I, Husain M, et al. Prevalence and social correlates of postnatal depression in a low income country. *Archives of women's mental health* 2006;9:197-202.
21. Husain N, Parveen A, Husain M, et al. Prevalence and psychosocial correlates of perinatal depression: a cohort study from urban Pakistan. *Archives of women's mental health* 2011;14:395-403.
22. Imran N, Haider II. Screening of antenatal depression in Pakistan: risk factors and effects on obstetric and neonatal outcomes. *Asia-Pacific Psychiatry* 2010;2(1):26-32.
23. Irum S, Khan AA, Rabbani U, et al. Frequency and Risk Factors of Anxiety and Depression among Pregnant Women in Abbottabad, Pakistan: A Facility-Based Cross-Sectional Study. *Pakistan Journal of Medical and Health Sciences* 2022;16(7):465-68. doi: https://dx.doi.org/10.53350/pjmhs22167465
24. Ishtiaque S, Sultana S, Malik U, et al. Prevalence of antenatal depression and associated risk factors among pregnant women attending antenatal clinics in Karachi, Pakistan. *Rawal Medical Journal* 2020;45(2):434-38.
25. Jabbar SA, Butt AS, Balouch AH, et al. Frequency of Depression in Pregnant Patients Presenting to Antenatal Clinic. A Cross-Sectional Study. *Pakistan Journal of Medical and Health Sciences* 2022;16(11):333-35. doi: https://dx.doi.org/10.53350/pjmhs20221611333
26. Jamal BA, Dastgir G, Khan MS, et al. Antenatal depression: Prevalence predictors and frequently employed coping strategies. *PAKISTAN JOURNAL OF MEDICAL & HEALTH SCIENCES* 2018;12(2):432-36.
27. Kalar MU, Fatima I, Nabila K, et al. Prevalence and predictors of postnatal depression in mothers of Karachi. *International Journal of Collaborative Research on Internal Medicine & Public Health* 2012;4(5):830-39.
28. Kalyani GHS. Incidence of depressive illness in Pakistani women during postnatal period. *JCPSP-Journal of the College of Physicians and Surgeons Pakistan* 2001;11(4):246-8.
29. Karmaliani R, Asad N, Bann CM, et al. Prevalence of anxiety, depression and associated factors among pregnant women of Hyderabad, Pakistan. *International Journal of Social Psychiatry* 2009;55(5):414-24.
30. Kazi A, Fatmi Z, Hatcher J, et al. Social environment and depression among pregnant women in urban areas of Pakistan: importance of social relations. *Social Science & Medicine* 2006;63(6):1466-76.
31. Khalid R. Maternity blues and puerperal depression in Pakistani women. *Pakistan Journal of Psychological Research* 1989;4(3-4):71-81.
32. Khan MJ, Hamza MA, Sarwar I, et al. Major Depressive Disorder: An Alarming Stigma Of Pregnant Women. *Journal of Ayub Medical College, Abbottabad : JAMC* 2020;32(2):244-49.
33. Khan R, Waqas A, Mustehsan ZH, et al. Predictors of Prenatal Depression: A Cross-Sectional Study in Rural Pakistan. *Frontiers in Psychiatry* 2021;12:584287. doi: https://dx.doi.org/10.3389/fpsyt.2021.584287
34. Khanam M, Sultana A, Siddiqui SH, et al. Screening for postpartum depression in recently delivered mothers. 2011
35. Khanam R, Applegate J, Nisar I, et al. Burden and risk factors for antenatal depression and its effect on preterm birth in South Asia: A population-based cohort study. *PloS one* 2022;17(2):e0263091. doi: https://dx.doi.org/10.1371/journal.pone.0263091
36. LeMasters K, Andrabi N, Zalla L, et al. Maternal depression in rural Pakistan: the protective associations with cultural postpartum practices. *BMC public health* 2020;20(1):68. doi: https://dx.doi.org/10.1186/s12889-020-8176-0

37. Maqbool S, Manzoor I, Farman T, et al. Impact of COVID-19 on mental health of pregnant women in Punjab, Pakistan. *Pakistan Journal of Medical and Health Sciences* 2022;16(10):166-69. doi: https://dx.doi.org/10.53350/pjmhs221610166
38. Maselko J, Bates L, Bhalotra S, et al. Socioeconomic status indicators and common mental disorders: evidence from a study of prenatal depression in Pakistan. *SSM-population health* 2018;4:1-9.
39. Maselko J, Hagaman AK, Bates LM, et al. Father involvement in the first year of life: Associations with maternal mental health and child development outcomes in rural Pakistan. *Social Science & Medicine* 2019;237 doi: https://dx.doi.org/10.1016/j.socscimed.2019.112421
40. Masood A, Musarrat R, Mazahir S, et al. STRESS, ANXIETY AND DEPRESSION IN WOMEN WITH PRIMIGRAVADA: A STUDY ON PAKISTANI WOMEN. *Khyber Medical University Journal* 2017;9(3)
41. Mir S, Karmaliani R, Hatcher J, et al. PREVALENCE AND RISK FACTORS CONTRIBUTING TO DEPRESSION AMONG PREGNANT WOMEN IN DISTRICT CHITRAL, PAKISTAN. *Journal of Pakistan Psychiatric Society* 2012;9(1)
42. Muneer A, Minhas FA, Nizami A, et al. Frequency and associated factors for postnatal depression. *J Coll Physicians Surg Pak* 2009;19(4):236-39.
43. Niaz S, Izhar N, Bhatti M. Anxiety and depression in pregnant women presenting in the OPD of a teaching hospital. *Pakistan Journal of Medical Sciences* 2004;20(2):117-19.
44. Noorullah A, Mohsin Z, Munir T, et al. Prevalence of paternal postpartum depression. *Pakistan Journal of Neurological Sciences (PJNS)* 2020;15(3):11-16.
45. Premji SS, Lalani S, Shaikh K, et al. Comorbid Anxiety and Depression among Pregnant Pakistani Women: Higher Rates, Different Vulnerability Characteristics, and the Role of Perceived Stress. *International journal of environmental research and public health* 2020;17(19) doi: https://dx.doi.org/10.3390/ijerph17197295
46. Rabia S, Nusrat U, Qazi S. Frequency and risk profiles associated with antenatal anxiety and depression in middle socioeconomic women. *Annals of Abbasi Shaheed Hospital and Karachi Medical & Dental College* 2017;22(2):88-96.
47. Rahman A, Iqbal Z, Harrington R. Life events, social support and depression in childbirth: perspectives from a rural community in the developing world. *Psychological medicine* 2003;33(7):1161-67.
48. Rahman A, Creed F. Outcome of prenatal depression and risk factors associated with persistence in the first postnatal year: prospective study from Rawalpindi, Pakistan. *Journal of affective disorders* 2007;100(1-3):115-21.
49. Ramji RS, Noori MY, Faisal A. POSTPARTUM DEPRESSION (PPD) AMONG WORKING AND NON-WORKING MOTHERS/WOMEN IN KARACHI, PAKISTAN. *Journal on Nursing* 2016;6(3)
50. Riaz S, Riaz MN. Prenatal psychiatric symptoms as predictors of postnatal anxiety and depression among primary and multi-gravida women. *JPMA The Journal of the Pakistan Medical Association* 2020;70(12(A)):2138-42. doi: https://dx.doi.org/10.5455/JPMA.14559
51. Sabir M, Nagi MLF, Kazmi TH. Prevalence of antenatal depression among women receiving antenatal care during last trimester of pregnancy in a tertiary care private institute of Lahore. *Pakistan Journal of Medical Sciences* 2019;35(2):527.
52. Sadaf M, Kazmi F, Malik SN. Antenatal screening for postpartum depression. *Journal of Rawalpindi Medical College* 2011;15(1)
53. Sadiq G, Shahzad Z, Sadiq S. Prospective study on prevalence and risk factors of post natal depression in Rawalpindi/Islamabad, Pakistan. *Rawal Med J* 2016;41(1):64-67.
54. Saeed A, Raana T, Saeed AM, et al. Effect of antenatal depression on maternal dietary intake and neonatal outcome: a prospective cohort. *Nutrition journal* 2016;15(1):1-9.
55. Shah SMA, Bowen A, Afridi I, et al. Prevalence of antenatal depression: comparison between Pakistani and Canadian women. *JPMA-Journal of the Pakistan Medical Association* 2011;61(3):242.

56. Shah S, Lonergan B. Frequency of postpartum depression and its association with breastfeeding: A cross-sectional survey at immunization clinics in Islamabad, Pakistan. *JPMA The Journal of the Pakistan Medical Association* 2017;67(8):1151-56.
57. Shahid A, Malik NI, Shahid F, et al. Psychosocial predictors of mental health among pregnant women. *Perspectives in Psychiatric Care* 2022;58(3):1071-76. doi: 10.1111/ppc.12900
58. Shaikh K, Premji SS, Rose MS, et al. The association between parity, infant gender, higher level of paternal education and preterm birth in Pakistan: a cohort study. *BMC pregnancy and childbirth* 2011;11:1-10.
59. Tariq N, Naeem H, Tariq A, et al. Maternal depression and its correlates: A longitudinal study. *JPMA The Journal of the Pakistan Medical Association* 2021;71(6):1618-22. doi: https://dx.doi.org/10.47391/JPMA.352
60. Waqas A, Raza N, Lodhi HW, et al. Psychosocial factors of antenatal anxiety and depression in Pakistan: is social support a mediator? *PloS one* 2015;10(1):e0116510.
61. Zahidie A, Kazi A, Fatmi Z, et al. Social environment and depression among pregnant women in rural areas of Sind, Pakistan. *JPMA-Journal of the Pakistan Medical Association* 2011;61(12):1183.
62. Zareen N, Majid N, Naqvi S, et al. Effect of domestic violence on pregnancy outcome. *J Coll Physicians Surg Pak* 2009;19(5):291-6.
